# E3 Ubiquitin Ligase Rsp5 Controls Lifestyle Transition and Pathogenicity in *Arthrobotrys oligospora*

**DOI:** 10.3390/jof12080597

**Published:** 2026-08-11

**Authors:** Xiqi Zhang, Hailong Pu, Runliu He, Yonglan Liu, Na Sha, Xin Wang

**Affiliations:** 1 State Key Laboratory for Conservation and Utilization of Bio-Resources in Yunnan, Yunnan University, Kunming 650091, China; zhangxiqi@stu.ynu.edu.cn (X.Z.);; 2 Yunnan Key Laboratory of Basic Research and Innovative Application for Green Biological Production, Yunnan University, Kunming 650091, China; 3 Southwest United Graduate School, Kunming 650092, China

**Keywords:** *Arthrobotrys oligospora*, AoRsp5, ESCRT machinery, MAPK signaling, ferroptosis-like cell death, nematodes

## Abstract

Fungi have evolved sophisticated mechanisms to survive in complex environments. Nematode-trapping fungi (NTF) sense and recognize nematode prey to transition from a saprophytic to predatory lifestyle, rendering them promising biocontrol agents against plant-parasitic nematodes. Ubiquitination is essential for eukaryotic cellular homeostasis, yet its regulation of NTF development and pathogenicity remains unclear. Here, we identify the conserved E3 ubiquitin ligase AoRsp5 as a critical factor for all major life stages of *Arthrobotrys oligospora*. Knockout of *rsp5* impairs Endosomal Sorting Complex Required for Transport (ESCRT)-mediated endocytosis and compromises plasma membrane integrity. These defects trigger disturbed cellular iron homeostasis and ferroptosis-like cell death, accompanied by global dysregulation of protein phosphorylation and ubiquitination as well as prominent suppression of MAPK cascades, which further attenuate virulence-associated signaling. Overall, our findings reveal a pivotal role for Rsp5-mediated ubiquitination in coordinating cellular homeostasis and developmental transitions in NTF.

## 1. Introduction

Plant-parasitic nematodes (PPNs) are major pathogens that cause devastating plant diseases and inflict substantial crop yield losses [1]. Nematode-trapping fungi (NTF) serve as potential biotic regulators of soil nematode population dynamics, capturing and infecting nematodes by differentiating specialized predatory structures, including adhesive hyphae, adhesive knobs, adhesive networks, and constricting rings [2]. Within this group, members of the genus *Arthrobotrys*, particularly *Arthrobotrys oligospora* Fresen, are the most widely distributed across soil ecosystems [3]. This species serves as a representative model strain for investigating the biocontrol potential and predator–prey interactions of NTF. The formation of its trapping structures is a defining feature of the morphogenetic transition from a saprophytic to predatory lifestyle, and a key survival strategy for environmental adaptation and nutrient acquisition [4]. Under limited nutrient conditions, external signals such as extracellular nematode-derived chemicals, environmental ammonia, and urea function as triggers that drive the developmental shift toward a predatory lifestyle [5,6,7]. Emerging evidence highlights the role of G protein-coupled receptors (GPCRs), MAPK, and cAMP-PKA cascades in the regulation of trapping structures [8,9,10]. In addition, microRNA-like RNAs (milRNAs) modulate lifestyle transitions via RNA interference pathways [11]. Importantly, in other fungal pathogens, post-translational modifications (PTMs) such as phosphorylation and ubiquitination act as conserved regulators of development, stress adaptation, and pathogenesis [12,13]. Although previous evidence has linked certain E3 ubiquitin ligases to the developmental transitions of NTF [14], the PTM networks and regulatory basis in mycelial growth and complex morphological differentiation remain poorly understood.

Ubiquitination is a highly conserved PTM in eukaryotes, mediating protein modification and functional regulation through a cascade involving E1 activating, E2 conjugating, and E3 ligase enzymes [15]. This ubiquitin–proteasome system (UPS) not only mediates substrate degradation via the 26S proteasome [16], but also regulates biological processes including cell cycle progression, signal transduction, DNA repair, membrane trafficking, and protein quality control [17,18,19]. E3 ubiquitin ligases confer substrate specificity and are categorized into three major classes: RING, HECT, and RBR types, and function as critical enzymes of the ubiquitination pathway [20]. In fungi, E3 ligases underpin growth, development, and virulence [21,22]. For instance, deletion of *UBI1* in *Cryptococcus neoformans* (Sanfelice) Vuill disrupts ribosome biogenesis and its virulence during host infection [23]. Similarly, the protein MoCand2 of *Magnaporthe oryzae* Couch & Kohn enhances fungal pathogenicity by repressing CRL ubiquitin ligase activity [24].

Rsp5 (Reverses SPT-phenotype protein 5), a HECT-type E3 ubiquitin ligase belonging to the Nedd4 family, is the sole Nedd4 family member in *Saccharomyces cerevisiae* (Desm.) Meyen [25]. It has a modular structure comprising an N-terminal C2 domain for membrane targeting, two tandem WW domains for substrate recognition, and a C-terminal HECT catalytic domain that mediates ubiquitin transfer [26]. As a pivotal mediator of eukaryotic endocytosis, Rsp5 interacts with ART (Arrestin-related trafficking adaptors) PPX (Y/F) motifs via its WW domains [27]. This interaction mediates K63-linked ubiquitination of diverse plasma membrane proteins [28,29], and triggers their endocytosis [30]. Beyond cargo marking, Rsp5-mediated ubiquitination is coupled with the recruitment of the Endosomal Sorting Complex Required for Transport (ESCRT) complex components, which coordinates membrane dynamics, vesicle biogenesis, and endosomal sorting [31,32]. In *M. oryzae*, the ESCRT machinery is also involved in vegetative growth, conidiation, pathogenicity, abiotic stress responses, and endocytosis [33]. This conserved Rsp5-ESCRT further mediates vacuolar degradation, thereby maintaining cellular proteostasis and signal transduction [30,34]. Apart from its endocytic functions, Rsp5 regulates diverse cellular processes including fatty acid metabolism [35], mitochondrial protein import [36], clearance of aberrant nascent polypeptides [37], and RNA polymerase II degradation [38]. In the NTF *A. oligospora*, the Rsp5 ortholog is encoded by *AOL_s00188g58*. However, its specific contributions to the fungal lifestyle transition and pathogenic development remain largely uncharacterized.

Beyond its established role as a signaling molecule modulating fungal morphogenesis and virulence, ammonia has emerged as a critical environmental cue that initiates the ubiquitination pathway [6,39]. Despite these advances, the core regulatory role of ubiquitination in ammonia-mediated fungal lifestyle transition remains poorly defined. In this study, we aimed to elucidate the biological function and regulatory roles of the HECT-type E3 ubiquitin ligase AoRsp5 in *A*. *oligospora*. We show that ammonia-induced trap formation relies on a systematic shift in the ubiquitination regulatory network, which is controlled by AoRsp5. In *A. oligospora*, AoRsp5 plays an indispensable role in vegetative growth and the transition from vegetative growth to trap formation. This E3 ligase is required to preserve normal function of the ESCRT machinery for membrane protein endocytic trafficking, which helps maintain cellular proteostasis and major signaling pathways. Knockout of *rsp5* is associated with iron dyshomeostasis and the emergence of ferroptosis-like cell death. These cellular abnormalities are accompanied by reduced activity of the MAPK signaling cascade. Altogether, this work reveals diverse biological roles of AoRsp5-mediated ubiquitination in nematode-trapping fungi, and provides clues for understanding how cellular proteostasis and environmental signals interact to regulate fungal lifestyle transitions.

## 2. Materials and Methods

### 2.1. Fungal Strains and Nematode Cultivation

Wild-type (WT) *Arthrobotrys oligospora* ATCC 24927 was maintained on potato dextrose agar (PDA, 200 g potatoes, 20 g glucose, and 18 g agar). For selection and maintenance of transformants, the Δ*rsp5* mutant was grown on PDA supplemented with 100 μg/mL hygromycin B (MDBIO INC, Sugar Land, TX, USA, H004), while the complemented strain (*rsp5*-re) was cultured on PDA containing 100 μg/mL hygromycin B and 100 μg/mL nourseothricin (MKBio, Shanghai, China, MS0026). All cultures were incubated at 28 °C for 7 days [40]. The two antibiotics (hygromycin B, and nourseothricin) applied for transformant selection target the fungal ribosome and interfere with the protein synthesis pathway. Notably, all phenotypic and functional experiments in this study were performed on corresponding antibiotic-free culture media to rule out the impacts of antibiotics on fungal characteristics.

All strains used in this study were deposited in the Microbial Library of the Germplasm Bank of Wild Species in Southwest China. *Caenorhabditis elegans* (Maupas) was maintained and synchronized following standard protocols [41].

### 2.2. Sequence and Phylogenetic Analysis

Homologous sequences of Rsp5 and other E3 ubiquitin ligases from *A. oligospora* and other fungi were retrieved from NCBI and UniProt databases. Multiple sequence alignment was conducted in MAFFT v7.490. Using MEGA 11, we screened the optimal substitution model for amino acid sequences; gaps were removed by partial deletion with a 75% site coverage cutoff, with an initial neighbor-joining tree generated automatically. The JTT+G+I substitution model was selected according to the Bayesian Information Criterion (BIC). A maximum likelihood phylogenetic tree was constructed with four gamma rate categories using the NNI tree-search algorithm, and node support values were assessed with 1000 bootstrap replicates. Branches with bootstrap values ≥ 50% were regarded as well-supported. The phylogenetic tree was visualized and annotated with iTOL.

### 2.3. Transcriptome Sequencing and Data Analysis

To profile the transcriptional dynamics of *A. oligospora* in response to ammonia induction, WT mycelia were harvested at the pre-induction stage (Control) and four defined post-induction phases: induction (Am1), trap germination (Am2), trap growth (Am3) and maturation (Am4). To dissect the regulatory role of AoRsp5 in transcription, WT and Δ*rsp5* mycelia were collected after 7 days of cultivation in PDA liquid medium at 28 °C. All samples were immediately frozen in liquid nitrogen and stored at −80 °C. High-throughput RNA sequencing (RNA-seq) was performed by Majorbio Bio-pharm Technology Co., Ltd. (Shanghai, China), with bioinformatic analyses carried out on the Majorbio Cloud Platform (https://www.majorbio.com). The Benjamini–Hochberg method was applied to calculate FDR, and genes with FDR < 0.05 were defined as DEGs.

### 2.4. RNA Extraction and RT-qPCR Analysis

Total RNA was extracted from fungal hyphae with TRIzol reagent (Invitrogen, Carlsbad, CA, USA, 15596018) following the manufacturer protocol. First-strand cDNA was synthesized using the HiScript II First Strand cDNA Synthesis Kit (Vazyme Biotech, Nanjing, China, R212). Quantitative real-time PCR (RT-qPCR) was performed with ChamQ SYBR qPCR Master Mix (Vazyme Biotech, Q311) on a LightCycler^®^ 480 Instrument II (Roche Diagnostics, Rotkreuz, Switzerland). Relative transcript levels were normalized to the internal reference gene *tubulin* and calculated via the 2^−ΔΔCt^ method [40]. Primer sequences are listed in Supplementary Table S2.

### 2.5. Subcellular Localization and eGFP Labeling

To characterize the spatiotemporal expression and subcellular distribution of AoRsp5, an eGFP-*rsp5* fusion construct was generated. The native *rsp5* promoter and coding sequence (CDS) were amplified and cloned in-frame into the pCT74 expression vector to generate an N-terminal eGFP fusion cassette. The construct was introduced into the WT strain, with positive transformants selected on PDA supplemented with 100 μg/mL nourseothricin [9]. Correct in-frame fusion was verified by PCR and Sanger sequencing, and the subcellular localization of the eGFP-AoRsp5 fusion protein was assessed via fluorescence microscopy.

### 2.6. Targeted Mutagenesis of rsp5

To investigate the function of *rsp5* in *A. oligospora*, precise gene knockout of *AOL_s00188g58* (*rsp5*) was performed via CRISPR-Cas9 ribonucleoprotein (RNP)-mediated homology-directed repair (HDR) [42]. A specific sgRNA targeting the *rsp5* locus (5′-AGTACCTTCGAAGACAACCAGGG-3′) was designed using ChopChop. For the HDR template, upstream and downstream homology arms were amplified from *A. oligospora* genomic DNA, and the hygromycin B resistance cassette (*hph*) was amplified from plasmid pCSN44 (primers listed in Supplementary Table S2). These three fragments were assembled into the linearized pCE-Zero vector via homologous recombination and propagated in *Escherichia coli*. PCR was further carried out with this construct as template to obtain the repair template. Transformations were performed using a PEG/CaCl_2_-mediated protoplast protocol. The pre-assembled Cas9-sgRNA RNP complex and HDR template were co-transformed into *A. oligospora* protoplasts. Primary transformants were selected on TYGA medium containing 100 μg/mL hygromycin B, and putative knockout mutants were verified for correct target integration by PCR amplification and Sanger sequencing.

### 2.7. Strain Complementation

To confirm that the observed phenotypes were specific to *rsp5* knockout, a complementation strain (*rsp5*-re) was generated. The *rsp5* coding sequence (CDS) and its native promoter were amplified from *A. oligospora* cDNA and genomic DNA, respectively, and cloned into the linearized pCE-Zero vector. This construct also harbored a nourseothricin resistance cassette (*natR*) from the pCfB3052 plasmid. The resulting complementation vector was transformed into Δ*rsp5* protoplasts via PEG-mediated transformation. Positive transformants were initially selected on PDA supplemented with 100 μg/mL hygromycin B and 100 μg/mL nourseothricin. Precise integration of the complementation cassette was validated by 5′ and 3′ junction PCR.

### 2.8. Trap Induction and Pathogenicity Assays

To evaluate ammonia-induced trap formation, 4 mm diameter mycelial pads were inoculated in the center of 6 cm water agar (WA) plates and incubated at 28 °C for 3 days, followed by trap induction using aqueous ammonia. For nematode-induced trap formation, approximately 200 synchronized *C. elegans* individuals were inoculated onto parallel WA plates to trigger trap development in the WT and Δ*rsp5* strains. For both induction systems, trap numbers were quantified, and representative images were acquired via bright-field light microscopy at 24 and 48 h post-induction [8]. Fungal pathogenicity was further evaluated by measuring nematode capture efficiency of each strain.

### 2.9. Mycelial Growth and Abiotic Stress Tolerance Assays

For vegetative growth assays, 4 mm diameter mycelial plugs were inoculated at the center of 6 cm Petri dishes containing solid PDA, CMY (cornmeal yeast extract agar), TG (tryptone-glucose agar), or TYGA (TG medium supplemented with 0.5% yeast extract). Colony diameters were measured after 7 days of incubation at 28 °C. For conidiation analysis, conidia were harvested from 7-day-old CMY cultures by gentle washing with 5 mL of sterile double-distilled water (ddH_2_O). Conidial morphology was observed under bright-field microscopy. Ten microliters of conidial suspension was taken for counting under light microscopy, and three biological replicates were performed for each experiment.

For stress tolerance assays, 4 mm diameter mycelial plugs of WT and Δ*rsp5* strains were inoculated onto PDA medium supplemented with different stress agents, including cell wall disruptors (0.01–0.03% SDS, 50–150 μg/mL Congo red) and osmotic stressors (0.1–0.2 M NaCl). Colony diameters were measured after 5 days of incubation at 28 °C, and the relative growth inhibition (RGI) was calculated according to the following formula: RGI = [(control colony diameter − stressed colony diameter)/(control colony diameter − 4 mm)] × 100% [43]. All assays were performed with at least three independent biological replicates.

### 2.10. Staining and Microscopic Imaging Assays

For visualization of fungal subcellular structures and ferroptosis-related signals, established fluorescent staining protocols were used. Plasma membranes, endocytic vesicles, and vacuoles were stained with 20 μM FM4-64 (MedChemExpress, Monmouth Junction, NJ, USA, HY-103466) [9]. Cell walls, plasma membranes, and nuclei were stained with 0.1% Calcofluor White (CFW, Yuanye Bio-Technology, Shanghai, China, S26637) [8], 5 μM PKH26 (MedChemExpress, Monmouth Junction, NJ, USA, HY-D1451), and 0.5 μg/mL DAPI (MedChemExpress, Monmouth Junction, NJ, USA, HY-D2868), respectively. Lipid droplets were stained with 1 μM Nile red (Sigma-Aldrich, St. Louis, MO, USA, 72485). To evaluate lipid peroxidation and characterize ferroptosis-like features in fungal hyphae, lipid peroxidation was detected using the BODIPY 581/591 C11 staining kit (Beyotime Biotechnology, Shanghai, China, S0043), and ferroptosis-related fluorescent signals were examined with a commercial ferroptosis detection kit (Beyotime Biotechnology, Shanghai, China, C1190S). After all staining procedures, samples were washed twice with phosphate-buffered saline (PBS), and images were acquired on a Nikon N-SIM S structured illumination super-resolution microscope (Nikon Corporation, Tokyo, Japan).

For ultrastructural analysis, hyphae harvested at pre-induction and post-induction stages of trap formation triggered by either ammonia or nematodes were imaged via cryo-scanning electron microscopy (cryo-SEM). For transmission electron microscopy (TEM), hyphae cultured on PDA were fixed with a glutaraldehyde-based electron microscopy fixative, with imaging performed at the Kunming Institute of Zoology, Chinese Academy of Sciences.

### 2.11. Protein Extraction, Western Blot Analysis, ATP and MDA Lipid Peroxidation Quantification

Total protein was extracted from fungal hyphae using RIPA lysis buffer (Beyotime Biotechnology, Shanghai, China, P0013B) supplemented with a protease inhibitor cocktail (Roche, 11873580001) and 1 mM phenylmethylsulfonyl fluoride (PMSF, Beyotime Biotechnology, Shanghai, China, ST505). Protein concentrations were determined using the bicinchoninic acid (BCA) assay kit (Affinibody LifeScience, Wuhan, China, AFBCA-500T). Equal amounts of protein were separated by sodium dodecyl sulfate-polyacrylamide gel electrophoresis (SDS-PAGE) using one-step PAGE gels (Affinibody LifeScience, Wuhan, China, NSF10), transferred onto polyvinylidene difluoride (PVDF) membranes (Merck-Millipore, Billerica, MA, USA, IPVH00010), and processed via standard immunoblotting protocols. Primary antibodies used included anti-β-tubulin (ABclonal, Wuhan, China, AC026), anti-phospho-P38 MAPK, anti-phospho-P44/42 MAPK (Cell Signaling Technology, CST, Danvers, MA, USA, #4511 and #9101, respectively), anti-phosphotyrosine rabbit mAb, and anti-ubiquitin rabbit mAb (PTM Biolabs, Chicago, IL, USA, PTM-702RM and PTM-1106RM, respectively). Signals were detected with appropriate horseradish peroxidase (HRP)-conjugated secondary antibodies and enhanced chemiluminescence (ECL) reagents (Affinibody LifeScience, Wuhan, China, AIWB-006). Intracellular ATP levels were determined using the ATP Assay Kit (Beyotime Biotechnology, S0026), and lipid peroxidation was quantified by measuring malondialdehyde (MDA) with the MDA Assay Kit (Beyotime Biotechnology, S0043). Both assays were performed strictly according to the manufacturer’s protocols.

### 2.12. Absolute Quantification by Droplet Digital PCR (ddPCR)

Absolute quantification of target gene transcript copies was performed on the MicroDrop-ONE droplet digital PCR (ddPCR) system (Forevergen Biosciences, Guangzhou, China). Reaction mixtures were prepared with ddPCR Master Mix, specific primers, probes, and template cDNA. Droplet generation and PCR amplification were carried out according to the manufacturer’s protocol. Post-amplification, fluorescence signals were acquired, and absolute copy number concentrations of target cDNA were calculated via the system’s dedicated analysis software based on Poisson distribution. Primer sequences are listed in Supplementary Tables S2 and S3.

### 2.13. Statistical Analyses

GraphPad Prism version 9.5.1 (GraphPad Software, San Diego, CA, USA) was used for graph plotting and statistical analyses. All observational and quantitative data were analyzed using two-tailed unpaired Student’s *t*-tests or one-way analysis of variance (ANOVA) with Tukey’s multiple comparison test. Double-blinded analysis was adopted for trap counting and colony measurement; researchers performing quantification were blinded to sample genotype information. Differences with *p* < 0.05 were considered statistically significant. All experiments were performed with three independent biological replicates.

## 3. Results

### 3.1. The HECT-Type E3 Ligase AoRsp5 Is Important for Ammonia-Induced Fungal Trap Formation

**Figure 1:**
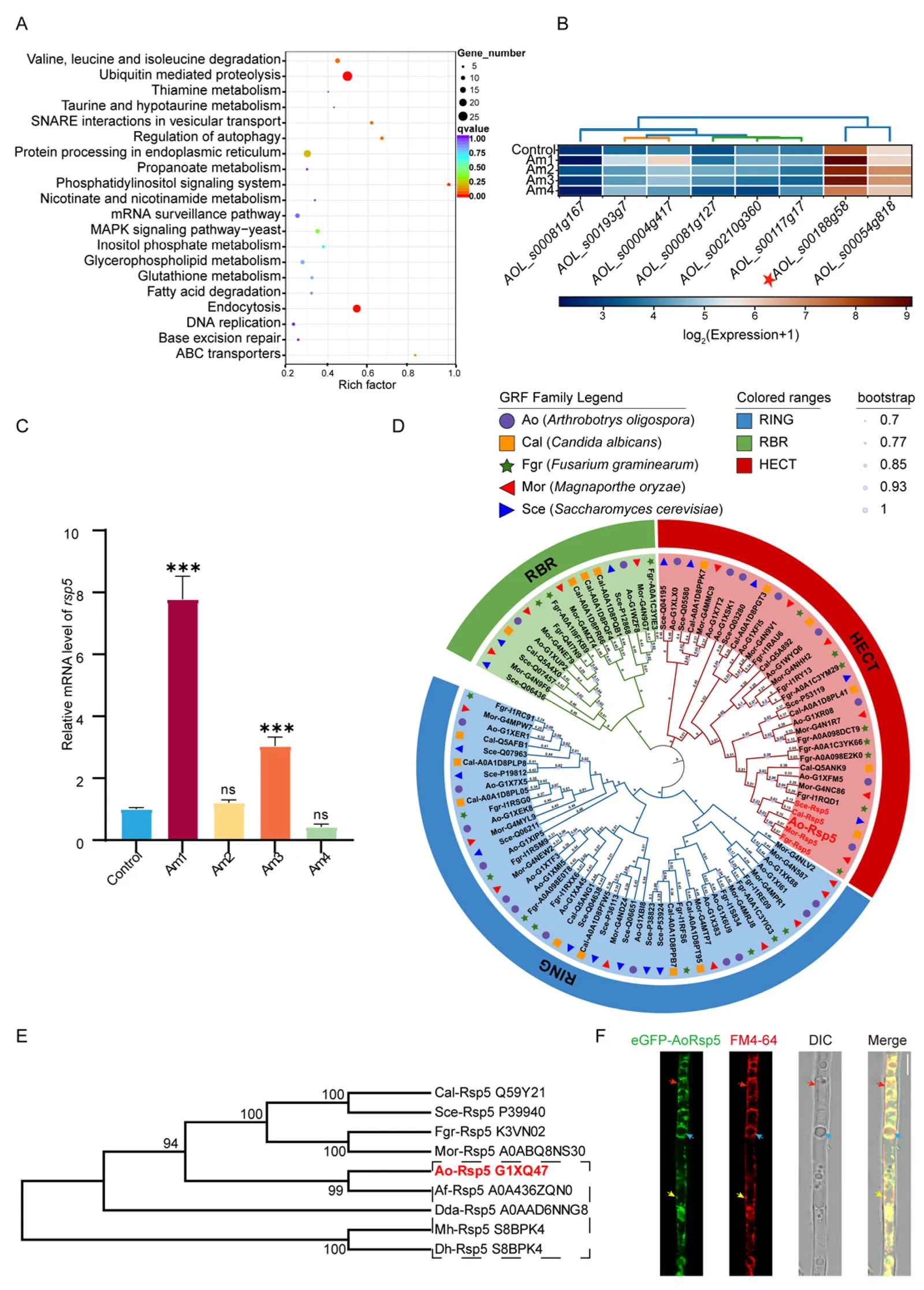
Identification of the HECT-type E3 ligase AoRsp5 involved in the ammonia response in *A. oligospora.* Fungal mycelia were harvested at the pre-induction stage (control) and four sequential post-induction stages of trap development: induction (Am1), trap germination (Am2), trap growth (Am3), and trap maturation (Am4). (**A**) KEGG pathway enrichment analysis of upregulated differentially expressed genes (DEGs) in the Am1 sample relative to the control. (**B**) Expression heatmap of all HECT-type E3 ubiquitin ligase family members in *A. oligospora*. *AOL_s00188g58* (rsp5) was indicated by a red asterisk. (**C**) Quantitative real-time PCR (RT-qPCR) validation of *rsp5* transcript levels during ammonia induction. *** *p* < 0.001; ns, not significant. All pairwise *p*-values were obtained via one-way ANOVA followed by Dunnett’s post hoc test. (**D**) Phylogenetic analysis of E3 ubiquitin ligase orthologs from *A. oligospora*, *C. albicans*, *F. graminearum*, *M. oryzae*, and *S. cerevisiae*. (**E**) Phylogenetic analysis of Rsp5 E3 ubiquitin ligase orthologs from *A. oligospora*, *Arthrobotrys flagrans* (Dudd.) Sidorova, Gorlenko & Nalepina, *Drechslerella dactyloides* (Drechsler) M. Scholler, Hagedorn & A. Rubner, *Monacrosporium haptotylum* (Drechsler) Xing Z. Liu & K.Q. Zhang, *Dactylellina haptotyla* (Drechsler) M. Scholler, Hagedorn & A. Rubner, *F. graminearum*, *M. oryzae*, *C. albicans*, and *S. cerevisiae*. Numbers on branches indicate bootstrap support values. Ao-Rsp5 (highlighted in red) was the protein characterized in this work, and each sequence was annotated with its UniProt accession number. The clade enclosed by the dashed box contains NTF. (**F**) Subcellular localization of the eGFP-AoRsp5 fusion protein in *A. oligospora*. Endosomes were indicated by yellow arrowheads, vacuolar membranes by blue arrowheads, and plasma membranes by red arrowheads. Mean Manders’ overlap coefficient = 0.817. Scale bar = 5 μm.

In previous studies, we identified that ammonia is a robust induction signal for the trap formation of the NTF *A. oligospora* [6,39]. To investigate the underlying signaling pathways, we analyzed RNA-seq data from mycelia exposed to ammonia, which revealed significant enrichment of the ubiquitination-mediated proteolysis and endocytosis pathways (Figure 1A). By profiling transcripts of the entire HECT-type E3 ubiquitin ligase family, *AOL_s00188g58* (hereafter designated *rsp5*) was identified as the top candidate, whose expression was strongly and specifically upregulated compared with other E3 ligases following ammonia treatment (Figure 1B). Further RT-qPCR verification revealed an approximately eight-fold increase in *rsp5* transcript levels in ammonia-treated mycelia (Figure 1C). Based on structural features and ubiquitin transfer mechanisms, E3 ubiquitin ligases are classified into three major families: RING, HECT, and RBR. AoRsp5 harbors a conserved HECT domain characteristic of HECT-type E3 ligases. Phylogenetic analysis and sequence alignment revealed that AoRsp5 shares high sequence similarity with Rsp5 orthologs from *Candida albicans* (C.P. Robin) Berkhout, *Fusarium graminearum* Schwabe, *M. oryzae*, *S. cerevisiae*, and other NTFs (Figure 1D−E and Figure S1A−C). To determine the subcellular localization of AoRsp5, we constructed an N-terminal eGFP fusion protein. Colocalization with the endocytic marker FM4-64 demonstrated that AoRsp5 predominantly localizes at endosomes, the plasma membrane (PM), and the vacuolar membrane (Figure 1F and Figure S3A,B). These results indicate that AoRsp5 functions as an endocytosis-associated E3 ligase involved in ammonia-induced trap formation.

### 3.2. AoRsp5 Is Critical for Hyphal Growth, Conidiation, and Trap Formation in A. oligospora

**Figure 2:**
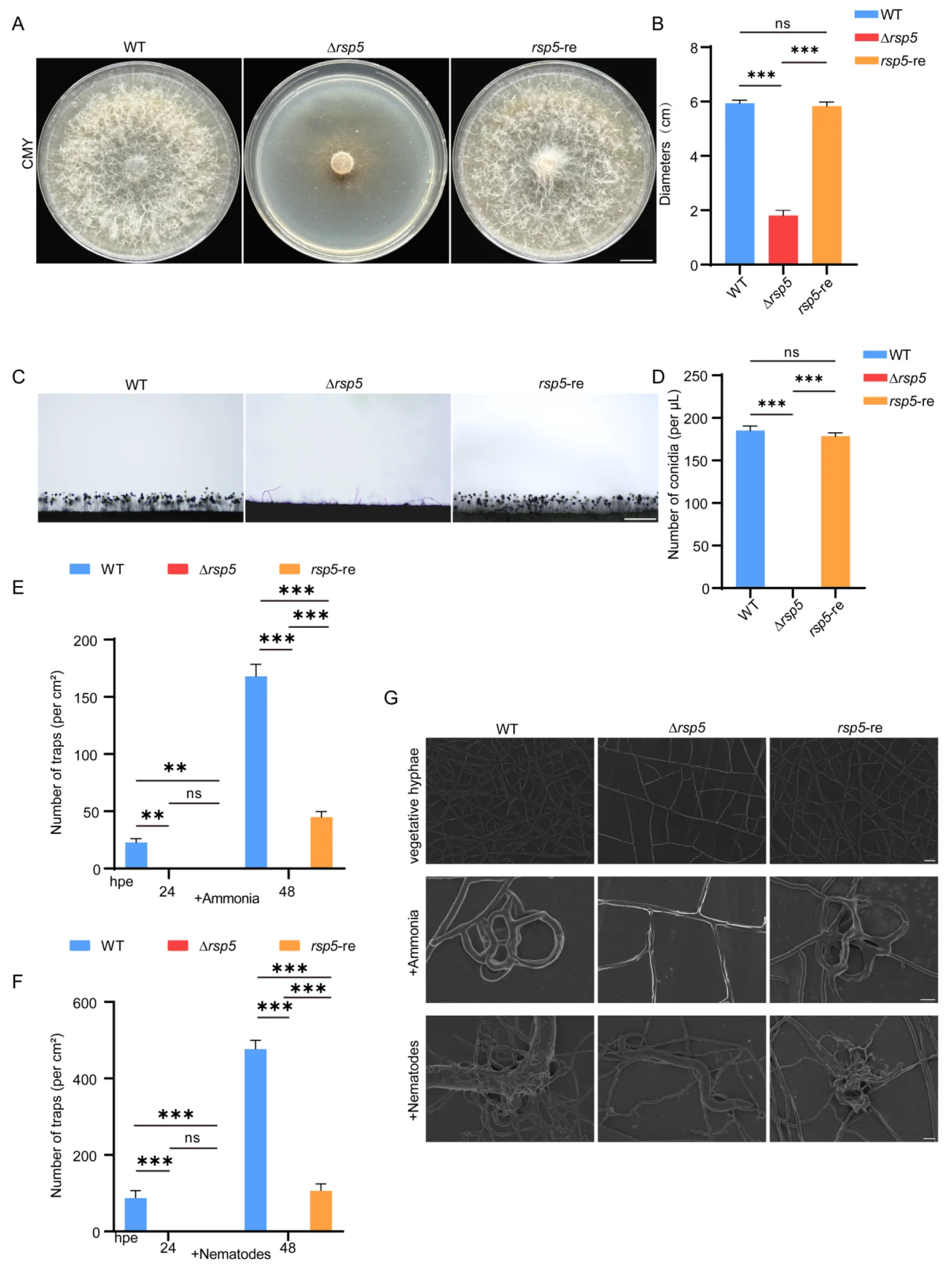
AoRsp5 is required for hyphal growth, conidiation, and trap formation in *A. oligospora.* (**A**) Hyphal growth of the Δ*rsp5* mutant strain. Δ*rsp5* colonies are indicated by white circles. Scale bar = 1 cm. (**B**) Statistical analysis of colony diameters of the indicated strains. (**C**) Microscopic observation of conidiophores in the indicated strains. Scale bar = 200 μm. (**D**) Quantification of conidial production in the indicated strains. (**E**,**F**) Quantification of trap formation in the indicated strains at different hours post exposure (hpe). (**G**) Scanning electron microscopy (SEM) observation of hyphae and traps. Scale bars = 50 μm, 10 μm, 20 μm. Statistical comparisons were performed via one-way ANOVA with Tukey’s post hoc test. *** *p* < 0.001, ** *p* < 0.01, ns, not significant.

To determine the regulatory role of AoRsp5, we generated *rsp5* knockout mutants via CRISPR-Cas9-mediated gene editing (Supplementary Figure S2A–D). The hyphal growth of the Δ*rsp5* mutant decreased significantly on all tested media. On CMY medium, the growth reduction reached approximately 69.67%, and conidiation was completely abolished (Figure 2A−D and Figure S3C−J), indicating that AoRsp5 is critical for vegetative growth in *A. oligospora*. We next examined its effect on the predatory phenotype. The Δ*rsp5* mutant completely lost the ability to form traps in response to either ammonia or nematodes (Figure 2E−G). A second independent knockout strain Δ*rsp5* #2 exhibited fully consistent phenotypes (Supplementary Figure S4). While the complemented strain completely restored conidiation capacity and fully rescued growth rate exclusively on CMY medium, it only partially recovered trap formation, and the number of traps produced was still significantly decreased compared with the wild-type strain (Figure 2 and Figure S3C−H). Scanning electron microscopy (SEM) revealed that knockout of *rsp5* resulted in shrunken and collapsed hyphal surfaces (Supplementary Figure S3I). These results demonstrate that the lifestyle transition of *A. oligospora* is strictly dependent on the HECT-type E3 ubiquitin ligase AoRsp5.

### 3.3. AoRsp5 Participates in Cellular Structure, Endocytic Trafficking, and ESCRT Machinery Regulation

**Figure 3:**
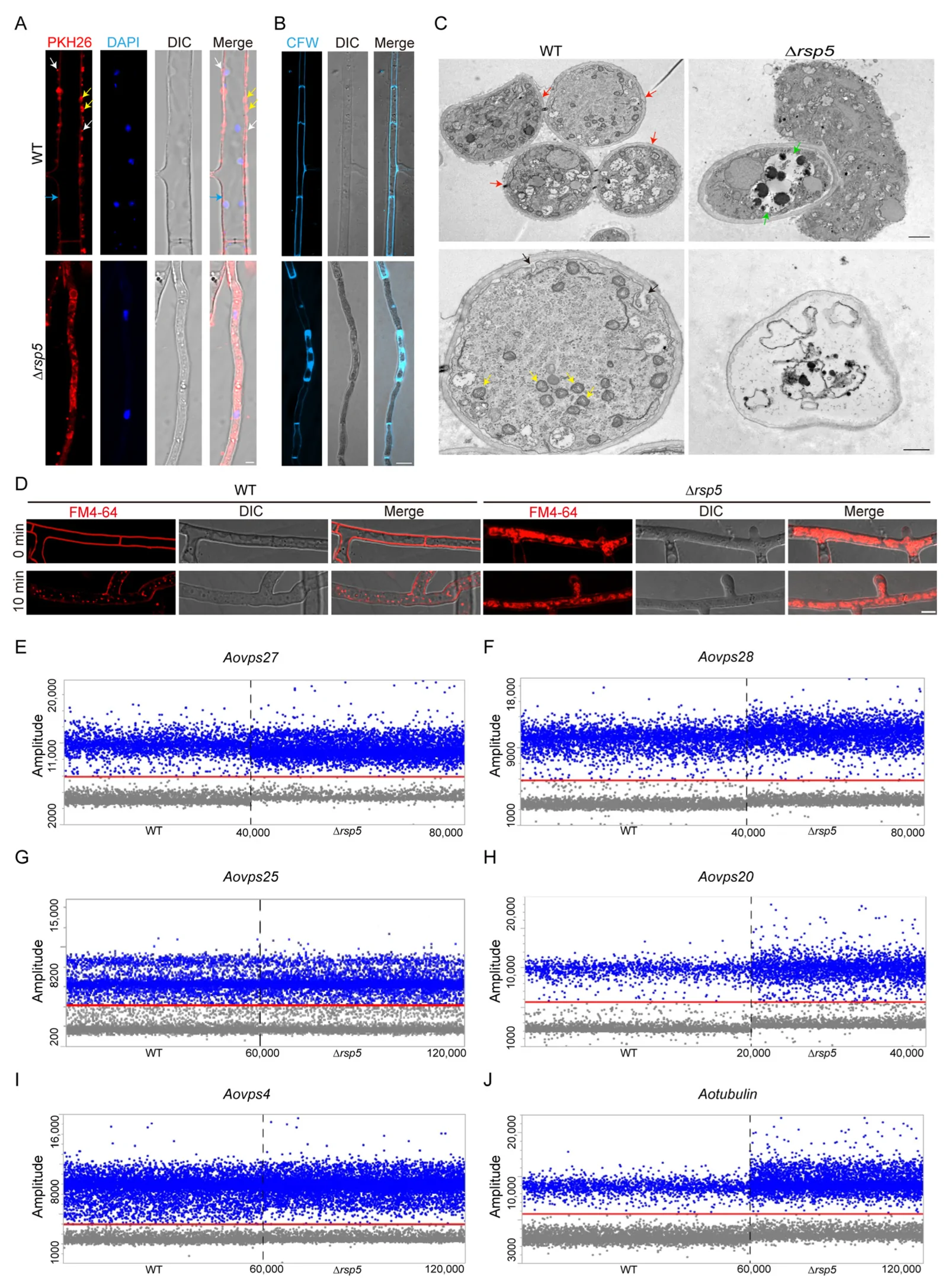
AoRsp5 is critical for plasma membrane integrity and ESCRT-mediated endocytic trafficking in *A. oligospora.* (**A**,**B**) Plasma membrane and cell wall staining with PKH26 and CFW, respectively. Endosomes are indicated by yellow arrows, vesicles by white arrows, and plasma membranes by blue arrows. Scale bars = 10 μm, 10 μm. (**C**) Ultrastructural observation of hyphae by transmission electron microscopy (TEM). Cell walls are indicated by red arrows, mitochondria by yellow arrows, endocytic structures by black arrows, and vacuoles by green arrows. Scale bars = 1 μm, 500 nm. ((**D**)) FM4-64 staining for endocytic trafficking assessment. Scale bar = 5 μm. (**E**–**J**) Absolute transcript quantification of genes encoding core ESCRT subunits and tubulin via ddPCR. Thresholds are indicated by red lines.

To determine the cellular structures altered by *rsp5* knockout, we examined the plasma membrane and cell wall using the lipophilic endocytic tracer PKH26 and Calcofluor White (CFW) staining. In the WT strain, PKH26 labeled regular vesicular morphology dispersed along the PM. In contrast, Δ*rsp5* hyphae exhibited an irregular accumulation of cytoplasmic fluorescence. CFW staining revealed abnormal clump-like aggregations in the mutant, accompanied by hyphal swelling and malformation (Figure 3A,B). Consistent with these observations, TEM analysis revealed multiple ultrastructural abnormalities in Δ*rsp5* mutants. Specifically, the mutant cell walls were significantly thinner, with reduced electron density and an indistinct layered structure. In addition, vacuolar degradation was disrupted by excessive aggregate accumulation, and vesicle and mitochondrial morphology were impaired in the Δ*rsp5* mutant (Figure 3C).

The ESCRT-dependent endocytic pathway is an important regulator linking the ubiquitination system to ESCRT-mediated sorting [44]. To investigate how the ubiquitin ligase AoRsp5 impacts the endocytic trafficking in *A. oligospora*, we performed FM4-64 fluorescence assays to monitor membrane internalization and vesicular transport. In WT hyphae, FM4-64 initially labeled only the PM region, whereas the Δ*rsp5* mutant showed diffuse cytoplasmic fluorescence and aberrant puncta signals, indicating impaired early endocytosis. After 10 min, the WT strain displayed typical endocytic internalization with uniform vesicular signals. In contrast, the mutant retained intense, disorganized fluorescence, reflecting defective vesicle sorting and endosomal formation (Figure 3D). A second independent knockout strain Δ*rsp5* #2 recapitulated these endocytic phenotypes (Supplementary Figure S8A).

In *A. oligospora*, the ESCRT machinery consists of 16 protein members. The absolute transcript quantification by ddPCR showed that knockout of *rsp5* led to elevated expression of core ESCRT genes, including *Aovps27*, *Aovps28*, *Aovps25*, *Aovps20*, and *Aovps4* (Figure 3E−I and Figure S5, and Supplementary Table S1), suggesting that the ESCRT machinery rapidly responds at the expression level to compensate for the deficiency of RSP5. Overall, our findings demonstrate that AoRsp5 plays a crucial role in maintaining cellular proteostasis via the ESCRT machinery.

### 3.4. AoRsp5 Contributes to the Maintenance of Lipid Homeostasis and Functional Lipid Metabolism in A. oligospora

**Figure 4:**
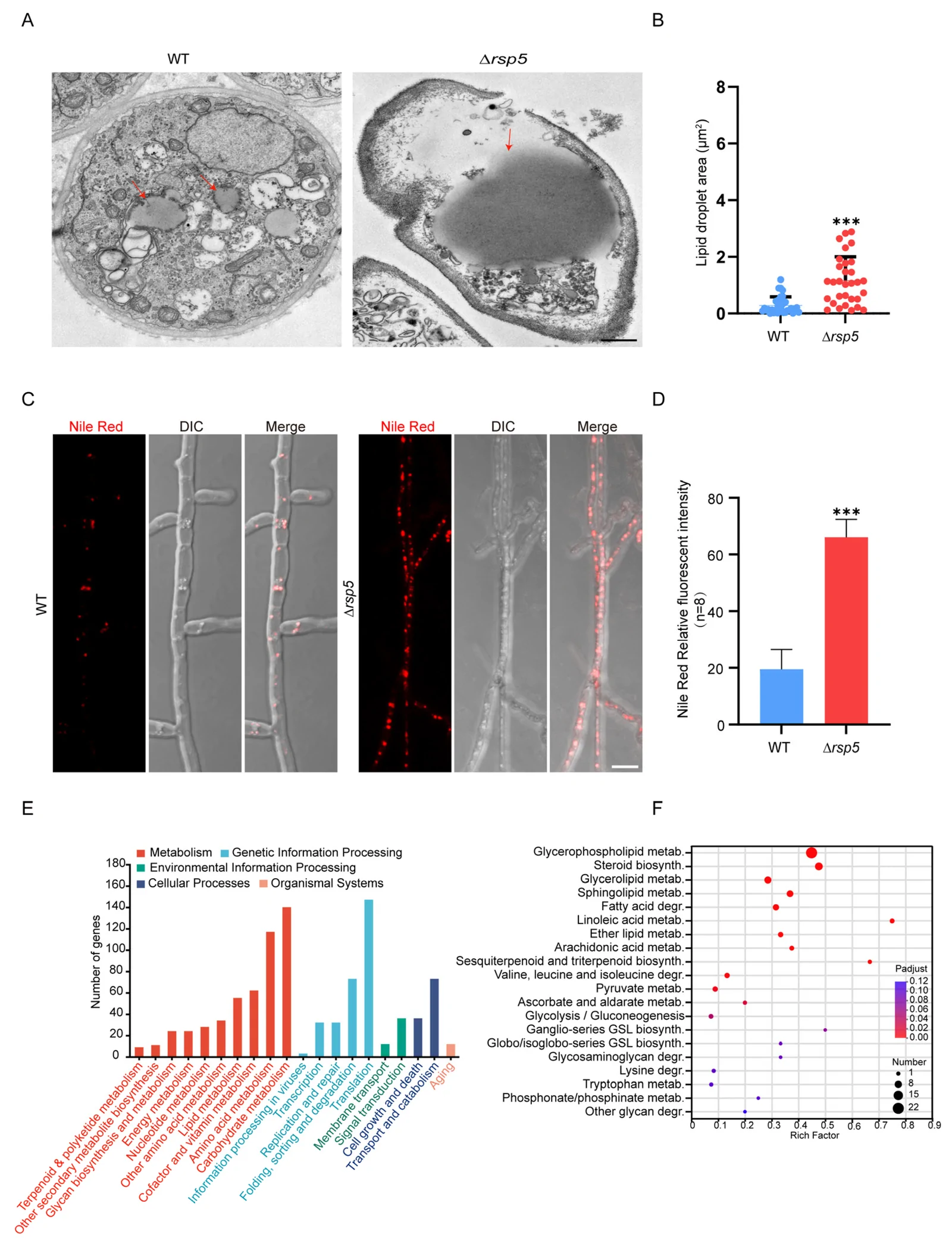
AoRsp5 mediates lipid metabolism in *A. oligospora.* (**A**) TEM analysis of lipid droplets. Lipid droplets are indicated by red arrows. Scale bar = 500 nm. (**B**) Lipid droplet area quantification. (**C**,**D**) Nile Red staining and fluorescence intensity analysis (*n* = 8 imaging fields from 3 biological replicates). (**E**,**F**) KEGG functional classification and pathway enrichment analysis of differentially expressed genes. Scale bar = 10 μm. *** *p* < 0.001. All *p*-values were calculated using an unpaired, two-tailed Student’s *t*-test.

Given that intact ESCRT trafficking and plasma membrane architecture are indispensable for balanced lipid metabolism, we first examined lipid storage morphology via TEM ultrastructural analysis. The results revealed prominent accumulation of enlarged lipid droplets in Δ*rsp5* mutants compared with the WT strain (Figure 4A,B). Nile Red neutral lipid staining further confirmed a significant elevation in intracellular neutral lipid content in the Δ*rsp5* mutant (Figure 4C,D). To further explore the underlying molecular mechanism, we performed transcriptome profiling (RNA-seq) on the wild-type and Δ*rsp5* mutant strains. KEGG functional category analysis showed a large proportion of differentially expressed genes (DEGs) from the Δ*rsp5* vs. WT comparison were annotated to the Metabolism and Genetic Information Processing categories (Figure 4E). Notably, within the metabolism category, lipid metabolism represented one of the markedly perturbed functional groups and was selected as the focus of subsequent analysis. Further KEGG pathway enrichment analysis demonstrated robust enrichment of multiple lipid-related pathways in the Δ*rsp5* mutant, particularly glycerophospholipid metabolism, steroid biosynthesis, glycerolipid metabolism, and sphingolipid metabolism (Figure 4F). These results support a critical role of AoRsp5 in maintaining global lipid homeostasis in *A. oligospora*.

### 3.5. AoRsp5 Contributes to the Regulation of Iron Homeostasis, Lipid Peroxidation and Ferroptosis-like Cell Death in A. oligospora

**Figure 5:**
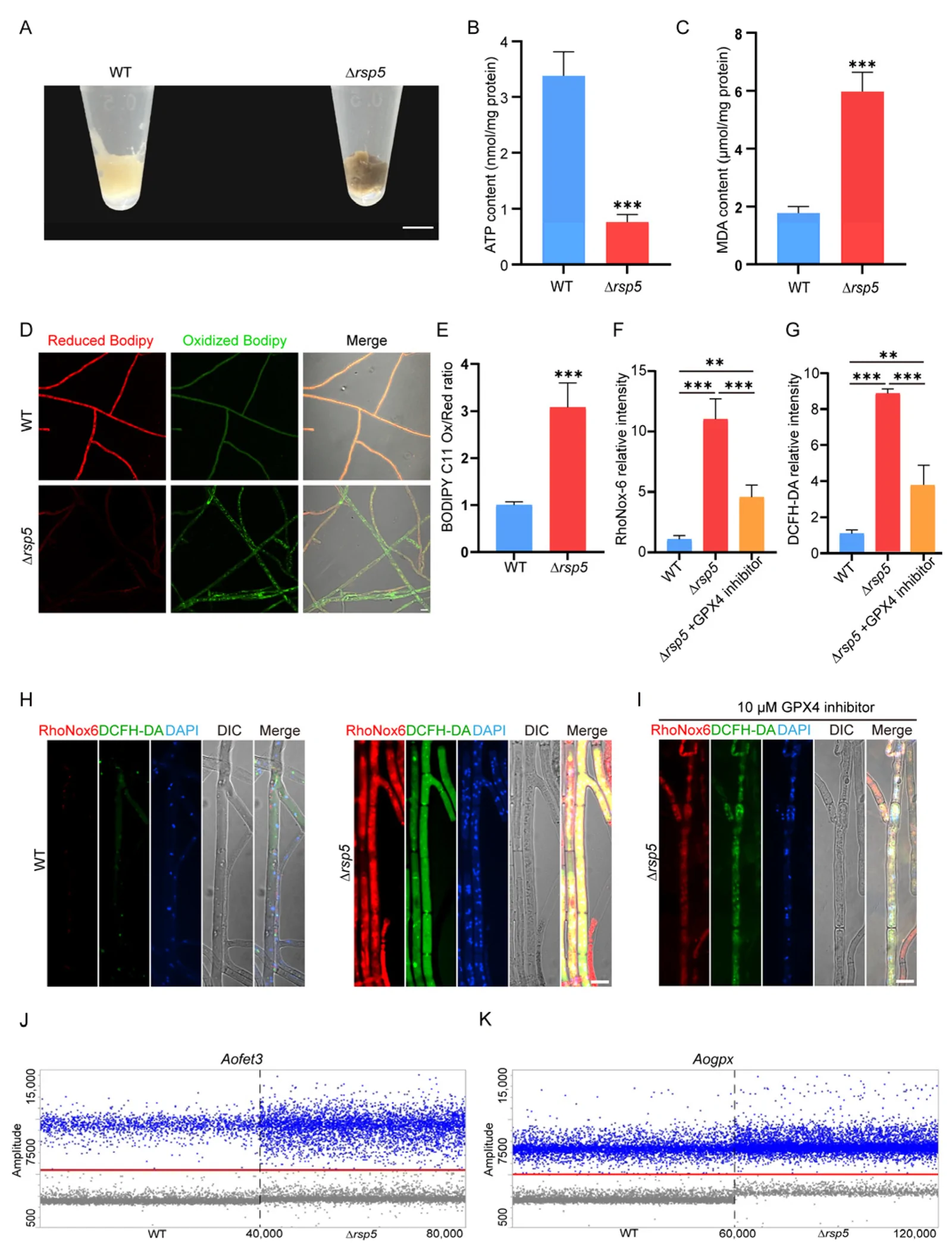
Disruption of AoRsp5 triggers ferroptosis-like cell death by perturbing iron homeostasis and inducing excessive lipid peroxidation in *A. oligospora*. (**A**) Hyphal pigmentation observation. Scale bar = 1 cm. (**B**) ATP content in WT and Δ*rsp5* strains. (**C**) MDA content in WT and Δ*rsp5* strains. (**D**,**E**) BODIPY C11 staining assay for lipid peroxidation in WT and Δ*rsp5* hyphae. Scale bar = 10 μm. (**F**–**I**) RhoNox6 (Fe^2+^) and DCFH-DA (ROS) dual staining combined with quantitative fluorescence analysis to characterize ferroptosis-like features in WT, Δ*rsp5*, and GPX4 inhibitor-treated Δ*rsp5* hyphae. Scale bar = 10 μm. (**J**,**K**) ddPCR-based absolute quantification of transcript levels for high-affinity iron transport gene *Aofet3* and antioxidant defense gene *Aogpx*. Thresholds are indicated by red lines. *** *p* < 0.001, ** *p* < 0.01. Statistical comparisons between two groups were performed using unpaired two-tailed Student’s *t*-test; statistical differences among three groups were determined by one-way ANOVA followed by Tukey’s multiple comparison test.

In our study, the Δ*rsp5* strain exhibited a brown pigmentation phenotype (Figure 5A). Ultrastructural analysis via TEM revealed severe mitochondrial damage in the Δ*rsp5* mutant, including distorted cristae and disorganized mitochondrial morphology (Figure 3C). Consistent with this mitochondrial dysfunction, ATP levels were significantly reduced in the Δ*rsp5* strain relative to the WT (Figure 5B). We further assessed lipid peroxidation levels using an MDA assay kit and BODIPY C11 staining, and the results showed that the Δ*rsp5* strain displayed markedly increased lipid peroxidation compared with the WT (Figure 5C,E).

To further verify the occurrence of ferroptosis-like cell death, we detected core ferroptosis-associated biochemical characteristics using a commercial ferroptosis detection kit. Under standard culture conditions, the WT strain exhibited weak RhoNox6 (red, Fe^2+^) and DCFH-DA (green, ROS) fluorescence, indicating low basal levels of intracellular labile iron and reactive oxygen species. In contrast, the two independent Δ*rsp5* knockout mutants (Δ*rsp5* and Δ*rsp5* #2) exhibited markedly elevated fluorescence signals for both probes, with strong signals distributed throughout the cytoplasm and plasma membrane. High-resolution imaging further revealed extensive colocalization of Fe^2+^ and ROS signals in the mutants (Figure 5F–H and Figure S8B), indicating that the loss of AoRsp5 function induces excessive accumulation of intracellular free ferrous iron and ROS.

Treatment with ferroptosis inhibitors failed to fully rescue these abnormal cellular phenotypes (Figure 5I), confirming that *rsp5* knockout causes irreversible cellular damage and metabolic dysfunction. Meanwhile, ddPCR analysis showed elevated transcript levels of genes responsible for high-affinity iron transport (*Aofet3*, *Aoftr1*) and antioxidant defense (*Aogpx*) in the Δ*rsp5* strain (Figure 5J–K and Figure S6A). These findings demonstrate that AoRsp5 functions as a critical regulator of iron homeostasis and redox balance in *A. oligospora*, and knockout of this gene induces severe lipid peroxidation that ultimately drives ferroptosis-like cell death.

### 3.6. AoRsp5 Modulates Environmental Fitness and Impacts Downstream MAPK Signaling Cascades

**Figure 6:**
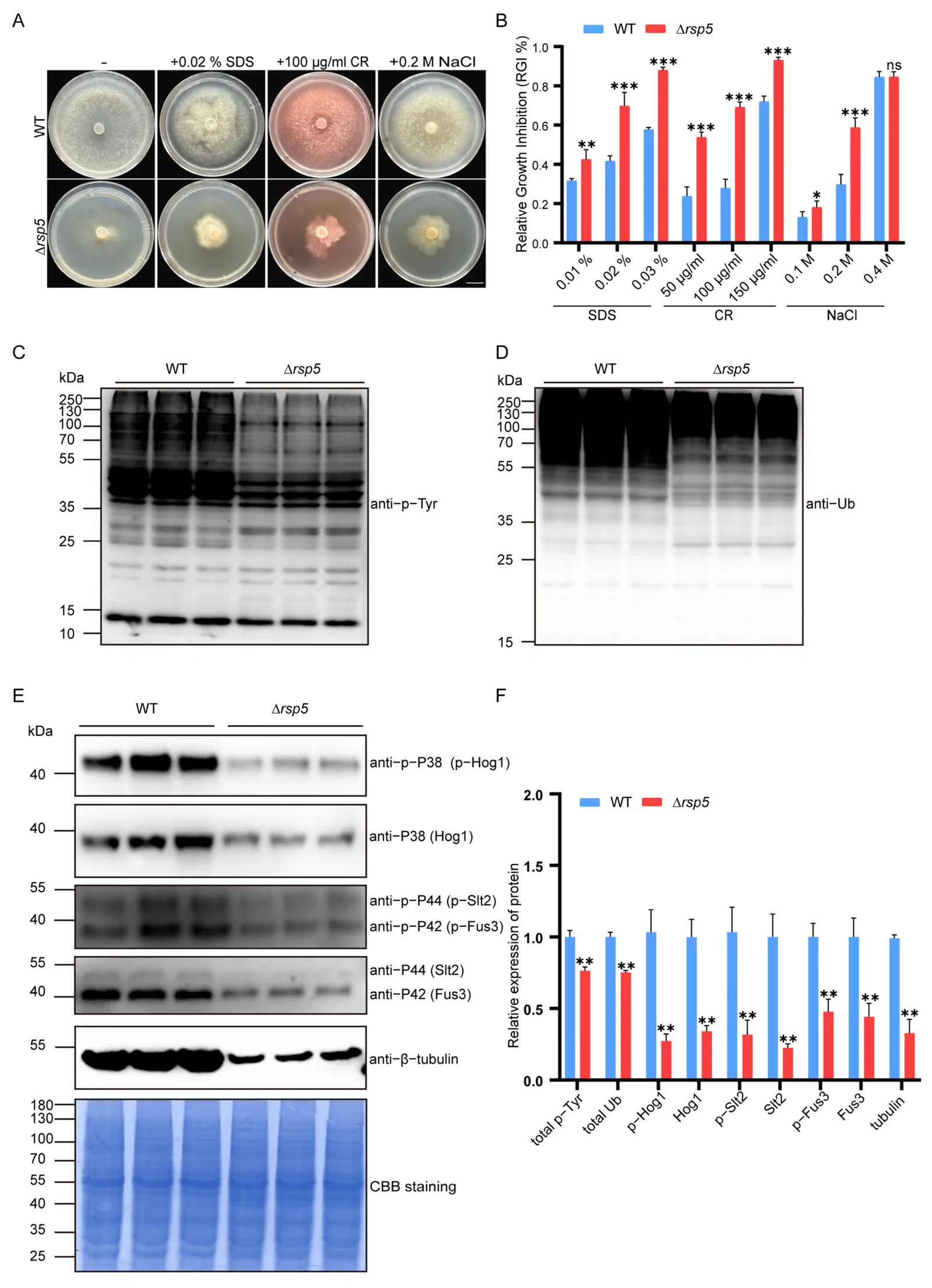
Knocking out *rsp5* compromises stress tolerance and indirectly dampens downstream MAPK cascades. (**A**,**B**) Stress sensitivity assays under cell wall and osmotic stress conditions. Scale bar = 1 cm. (**C**–**F**) Western blot analysis and quantification of total cellular phosphorylation, total ubiquitination, β-tubulin, as well as total protein and phosphorylation levels of core MAPK cascade components. Equal protein loading across lanes was verified by densitometry of total protein via Coomassie staining. * *p* < 0.05, ** *p* < 0.01, *** *p* < 0.001, ns, not significant. All raw *p*-values were calculated via unpaired two-tailed Student’s *t*-test, and corresponding *q*-values were generated after multiple testing correction.

Further, we performed stress sensitivity assays. The Δ*rsp5* mutant exhibited dose-dependent hypersensitivity to cell wall-damaging agents, including SDS (0.01–0.03%) and Congo Red (50–150 μg/mL), and showed significantly higher relative growth inhibition (RGI) than the WT strain at all tested concentrations. While both strains displayed impaired growth under NaCl-mediated osmotic stress, the Δ*rsp5* mutant was markedly more sensitive to low (0.1 M) and moderate (0.2 M) NaCl treatments. In contrast, no significant difference in osmotic tolerance was detected between the two strains under high-concentration (0.4 M) NaCl stress (Figure 6A,B). Consistently, the independent Δ*rsp5* #2 mutant exhibited identical stress sensitivity (Supplementary Figure S8C,D).

To elucidate the molecular basis of the above-mentioned phenotypes, we performed Western blotting to dissect the associated molecular mechanisms. We first assessed global protein post-translational modifications and found that total protein phosphorylation and ubiquitination levels were markedly reduced in the Δ*rsp5* mutant (Figure 6C,D). Meanwhile, the total protein abundance and activating phosphorylation levels of MAPK cascade components were drastically diminished in the Δ*rsp5* mutant (Figure 6E,F and Figure S7A,B), including Slt2 (cell wall integrity pathway), Fus3 (pheromone response pathway), and Hog1 (osmotic adaptation pathway). We further conducted absolute quantification via digital PCR to measure the transcript level of the non-canonical MAPK pathway effector *AoIme2* [45], which exhibited a striking downregulation in the Δ*rsp5* mutant (Supplementary Figure S6H and Supplementary Table S1). In addition, multiple housekeeping proteins conventionally used as internal references, such as β-tubulin, β-actin, and GAPDH, displayed reduced expression and thus could not serve as reliable loading controls for normalization (Supplementary Figure S7C). We also detected an aberrant double band of histone H3 specifically in the Δ*rsp5* mutant (Supplementary Figure S7C). Collectively, these lines of evidence demonstrate that knockout of rsp5 severely disrupts cellular proteostasis, accompanied by suppression of the MAPK signaling pathway.

## 4. Discussion

Ubiquitination functions as a sophisticated regulatory mechanism in fungi, dynamically modulated by intrinsic signaling pathways and extrinsic environmental cues. This post-translational modification (PTM) governs essential biological processes, including fungal growth and pathogenesis, and represents a pivotal intervention target for controlling dimorphism and virulence in *F. graminearum* [46]. In NTF, the formation of specialized predatory structures, which constitutes the critical stage of host infection, is strictly coordinated by the integration of nutritional and host-derived signals. This research demonstrates that ammonia-induced adhesive trap formation in *A. oligospora* is highly dependent on the ubiquitination system. AoRsp5 emerges as a pleiotropic E3 ligase important for the developmental and metabolic homeostasis of *A. oligospora*. Knockout of *rsp5* impairs ESCRT-mediated endocytosis and plasma membrane integrity in *A. oligospora*. The Δ*rsp5* mutant exhibits severe ferroptosis-like cell death, accompanied by reduced global ubiquitination and protein phosphorylation. We detected significantly decreased MAPK signaling activity, leading to abolished trap formation, as well as severe defects in vegetative growth and stress tolerance. This work suggests a close functional link between AoRsp5-mediated ubiquitination, cell survival, and MAPK signaling in NTF.

### 4.1. Evolutionary Conservation and Functional Analysis of AoRsp5

In *A. oligospora*, AoRsp5 regulates multiple critical cellular processes, including vegetative growth, conidiation, and trap formation. Phylogenetic analysis reveals that AoRsp5 is highly conserved among homologs from *C. albicans*, *F. graminearum*, *M. oryzae*, and *S. cerevisiae*, all of which belong to the conserved HECT-type E3 ubiquitin ligase family (Figure 1D,E and Figure S1A). In these fungi, *rsp5* typically serves as a housekeeping gene, and its knockout is invariably lethal. For instance, Rsp5 cooperates with the arrestin Rod1 to promote transporter endocytosis in yeast, yet rsp5 knockout is lethal [47]. In *F. graminearum*, the lethality of *rsp5* mutations has hampered functional studies, with Rsp5 only identified as a potential interacting partner of the acetyltransferase Gcn5 [46]. While another UBR1 E3 ligase mediates the predatory transition via the N-end rule pathway in *A. oligospora*, its deletion only decreases trap formation by 40% [14]. Notably, our Δ*rsp5* mutant exhibits an “all-or-nothing” phenotype. The mutant shows severe defects in vegetative growth and conidiation, and is completely unable to form traps in response to ammonia or nematodes (Figure 2, Figures S3 and S4). Knockout of *rsp5* also strongly represses transcription of the conidiation cascade *brlA–abaA–wetA* (Supplementary Figure S6B–G) [48]. These findings identify AoRsp5 as a functionally conserved survival regulator in *A. oligospora*.

### 4.2. AoRsp5 Is Critical for ESCRT-Mediated Plasma Membrane Quality Control

The Rsp5-ESCRT regulatory module is highly conserved across eukaryotes, where Rsp5 deficiency typically triggers aberrant PM protein accumulation, compensatory ESCRT stress responses, and abnormal endosomal enlargement [27,34,49]. Our study demonstrates that FM4-64 staining of Δ*rsp5* hyphae reveals diffuse fluorescence and intracellular dye retention (Figure 3D and Figure S8A). Consistent with these observations, *rsp5* knockout induces compensatory upregulation of ESCRT complex subunits (Figure 3E–I and Figure S5, and Supplementary Table S1), establishing AoRsp5 as a critical regulator of the ESCRT machinery. These findings confirm the conserved role of the Rsp5-mediated ESCRT machinery in maintaining endosomal sorting, intracellular trafficking, and PM homeostasis in NTF.

In yeast, the Rsp5–ART (arrestin-related trafficking adaptor) network constitutes the eukaryotic plasma membrane quality control (PMQC) system. By mediating the degradation of misfolded proteins via the endocytosis–ESCRT pathway, this network prevents misfolded protein accumulation and preserves membrane integrity [50]. The aberrant FM4-64 staining pattern in Δ*rsp5* mutants highlights systemic disruption of the membrane trafficking network (Figure 3D and Figure S8A). Within the mutant, the dye non-specifically enters the cytoplasm through a compromised plasma membrane, while endosomes fail to properly sort to the vacuole, leading to cytosolic dye leakage. Such a staining profile is consistent with that observed in *Schizosaccharomyces pombe* Lindner ESCRT mutants, such as Δ*sst6*, Δ*vps24*, and Δ*sst4* [51], as well as the Δ*vps24*, Δ*vps35*, and Δ*vps41* knockout strains in *A. oligospora* [52,53].

Loss of *rsp5* causes an overall decrease in cellular ubiquitination, directly impairing modification of ESCRT subunits [54]. Although transcriptionally upregulated, ESCRT proteins fail to assemble into functional complexes, rendering the compensatory response ineffective. This activation represents a cellular stress response to PMQC failure, in which elevated ESCRT expression attempts to overcome defective substrate sorting caused by weak ubiquitin signals. Rsp5-mediated K63-linked ubiquitination of membrane proteins acts as a key signal for ESCRT recruitment to endosomes [28,50]. Ubiquitinated cargo is subsequently recognized by the ubiquitin-binding domains (UBDs) of ESCRT-0/I, initiating multivesicular body (MVB) sorting [32,55]. These lines of evidence support a model where AoRsp5 functions as the ortholog of Sce-Rsp5, acting as a key component of the plasma membrane quality control (PMQC) system in NTF.

### 4.3. Dysregulation of the AoRsp5–ESCRT Pathway Triggers Ferroptosis-like Cell Death

This study demonstrates that knockout of *rsp5* induces ferroptosis-like cell death in *A. oligospora* (Figure 5). In *S. cerevisiae*, Rsp5 mediates substrate ubiquitination for subsequent ESCRT complex recognition and vacuolar degradation, thereby regulating intracellular iron transport [56]. Under iron-replete conditions, the high-affinity iron complex Fet3p–Ftr1p undergoes ubiquitination and subsequent turnover mediated by Rsp5. Knockout of *rsp5* causes this complex to evade ubiquitination and persistently accumulate at the plasma membrane, driving excessive iron uptake [57]. Beyond this post-translational control, Rsp5 can regulate transcriptional homeostasis of *fet3* and *ftr1* by promoting ubiquitin-dependent degradation of the iron-responsive transcription factor Aft1p [58]. In our absolute quantification assays, the transcript levels of *fet3* and *ftr1* are upregulated in the Δ*rsp5* mutant (Figure 5J,K and Figure S6A). This may be because impaired ubiquitination of the Fet3p–Ftr1p complex hinders recognition by the ESCRT machinery and further exacerbates intracellular iron accumulation. Previous work from our group established that *A. oligospora* utilizes PKS-derived metabolites for iron efflux and extracellular Fenton reactions, while NRPS siderophores recycle iron to limit intracellular reflux [59,60,61]. Dysregulation of the AoRsp5-ESCRT pathway may ultimately lead to uncontrolled intracellular Fenton reactions, excessive accumulation of reactive oxygen species (ROS), and widespread lipid peroxidation of the plasma membrane, thereby inducing ferroptosis-like cell death in the Δ*rsp5* mutant.

### 4.4. Indirect MAPK Signaling Perturbation Following rsp5 Deletion

It has been identified that RSP5 functions as a negative regulator of the MAPK signaling pathway through ubiquitin-mediated protein degradation. Studies in *S. cerevisiae* have verified the conserved repressive function of Rsp5 against MAPK cascades. Rsp5 ubiquitinates the plasma membrane sensor Msb2p to trigger ESCRT-dependent endosomal sorting and dampen MAPK signaling [62]. Further research reveals that Rsp5 also targets the upstream small GTPase Cdc42 for ubiquitin-mediated degradation. Abnormal accumulation of Cdc42 leads to hyperactivation of the MAPK pathway [63]. Similarly, the E3 ligase Pub1 in *S. pombe* negatively regulates stress responses by targeting components of the Spc1 MAPK cascade [64]. In plants, E3 ubiquitin ligases typically repress MAPK-dependent immune signaling by degrading positive regulators such as MKK4 and MKK5 [65]. Conserved Rsp5-mediated crosstalk between endocytic membrane transport and MAPK cascades has also been characterized in multiple filamentous fungi. In *M. oryzae*, the endosomal–vacuolar transport system serves as the essential docking platform for Pmk1 MAPK activation [66], and loss of the endocytic regulator MoEnd3 blocks receptor internalization, attenuates MAPK signaling, and impairs appressorium development and virulence [67]. In *Aspergillus nidulans* (Eidam) G. Winter, the core AnSte11–Ste50–Ste7–Fus3 MAPK module relies on intact plasma membrane–endosome trafficking cycles to drive developmental and secondary metabolic programs; disrupted membrane transport abolishes MAPK nuclear translocation and downstream gene expression [68].

In our study, knockout of *rsp5* disrupts ESCRT-mediated endocytosis, compromises plasma membrane integrity, and induces ferroptosis-like cell death in *A. oligospora*, accompanied by reduced global ubiquitination and protein phosphorylation, as well as indirect suppression of MAPK signaling. Given that Rsp5 typically ubiquitinates membrane receptors, especially GPCRs [69], we speculate that knockout of rsp5 impairs the trafficking of upstream sensory receptors to late endosomes, which in turn attenuates MAPK signaling activity. This model is merely put forward as a hypothetical explanation for the observed signaling changes, and we will fully dissect the complete regulatory cascade in future investigations.

## 5. Conclusions

Our study identifies the conserved E3 ubiquitin ligase AoRsp5 as an important factor for the vegetative growth and predatory capacity of *A. oligospora*. Upon loss of *rsp5*, the strains exhibit multiple abnormalities including defective ESCRT-dependent sorting, compromised plasma membrane integrity, disrupted iron homeostasis, and ferroptosis-like cell death. These perturbations are coupled with reduced MAPK signaling activity and impaired virulence. Our findings have expanded the known regulatory landscape of ubiquitination in fungal development and cellular metabolic homeostasis. Despite this, the full repertoire of direct ubiquitination substrates of AoRsp5 remains to be defined. Future work combining ubiquitinomic and interactomic screenings will characterize its direct substrates and the underlying regulatory networks.

## Data Availability

The data for this study, both within the article and Supplementary Materials, are readily accessible. All supporting data are provided in the Supplementary Figures S1–S8 and Supplementary Tables S1–S3.

## Supplementary Materials

The following supporting information can be downloaded at: https://www.mdpi.com/article/10.3390/jof12080597/s1, Figure S1: Sequence conservation and domain architecture of AoRsp5; Figure S2: CRISPR-Cas9 ribonucleoprotein (RNP)-mediated homology-directed repair (HDR) for targeted deletion of *rsp5* and validation of the knockout strain; Figure S3: Subcellular localization of AoRsp5 and phenotypic characterization of the Δ*rsp5* mutant in *A. oligospora*; Figure S4: Phenotypic characterization of a second independent Δ*rsp5* strain (Δ*rsp5* #2) of *A. oligospora*; Figure S5: Digital PCR validation of core ESCRT pathway genes in the WT and Δ*rsp5* strains; Figure S6: Digital PCR absolute transcript quantification of iron metabolism, sporulation-related, and non-canonical Ime2-type MAPK genes in WT and Δ*rsp5* strains; Figure S7: Total protein profiling and stress responses of WT and Δ*rsp5* strains; Figure S8: Phenotypic characterization of FM4-64 staining, ferroptosis, and stress resistance in the second independent Δ*rsp5* knockout strain (Δ*rsp5* #2) of *A. oligospora*; Table S1: Absolute quantification of gene expression in *A. oligospora* WT and Δ*rsp5* strains by ddPCR; Table S2: Primers list; Table S3: Primers list.

## Abbreviations

The following abbreviations are used in this manuscript:

NTF	Nematode-trapping fungi
ESCRT	Endosomal Sorting Complex Required for Transport
PPNs	Plant-parasitic nematodes
GPCRs	G protein-coupled receptors
milRNAs	microRNA-like RNAs
PTMs	post-translational modifications
UPS	ubiquitin–proteasome system
ART	Arrestin-related trafficking adaptors
RNA-seq	RNA sequencing
RNP	Ribonucleoprotein
HDR	homology-directed repair
hph	hygromycin B
CDS	coding sequence
natR	nourseothricin
WA	water agar
PDA	Potato Dextrose Agar
CMY	cornmeal yeast extract agar
TG	tryptone-glucose agar
TYGA	TG medium supplemented with 0.5% yeast extract
ddH_2_O	double-distilled water
RGI	relative growth inhibition
cryo-SEM	cryo-scanning electron microscopy
TEM	Transmission electron microscopy
PMSF	Phenylmethylsulfonyl fluoride
BCA	Bicinchoninic Acid
SDS-PAGE	sodium dodecyl sulfate-polyacrylamide gel electrophoresis
PVDF	polyvinylidene difluoride
ECL	enhanced chemiluminescence
MDA	malondialdehyde
ddPCR	droplet digital PCR
CFW	Calcofluor White
DEGs	differentially expressed genes
PMQC	plasma membrane quality control system
UBDs	ubiquitin-binding domains
MVB	multivesicular body
ROS	reactive oxygen species
