# Supplementary material for: E3 Ubiquitin Ligase Rsp5 Controls Lifestyle Transition and Pathogenicity in Arthrobotrys oligospora
Source: J Fungi (Basel). 2026 Aug 11;12(8):597. doi: 10.3390/jof12080597 (PMC13514188; doi:10.3390/jof12080597)
Supplement: Supplementary file 1 [file jof-12-00597-s001.zip › Supplementary Materials Xin Wang.pdf]

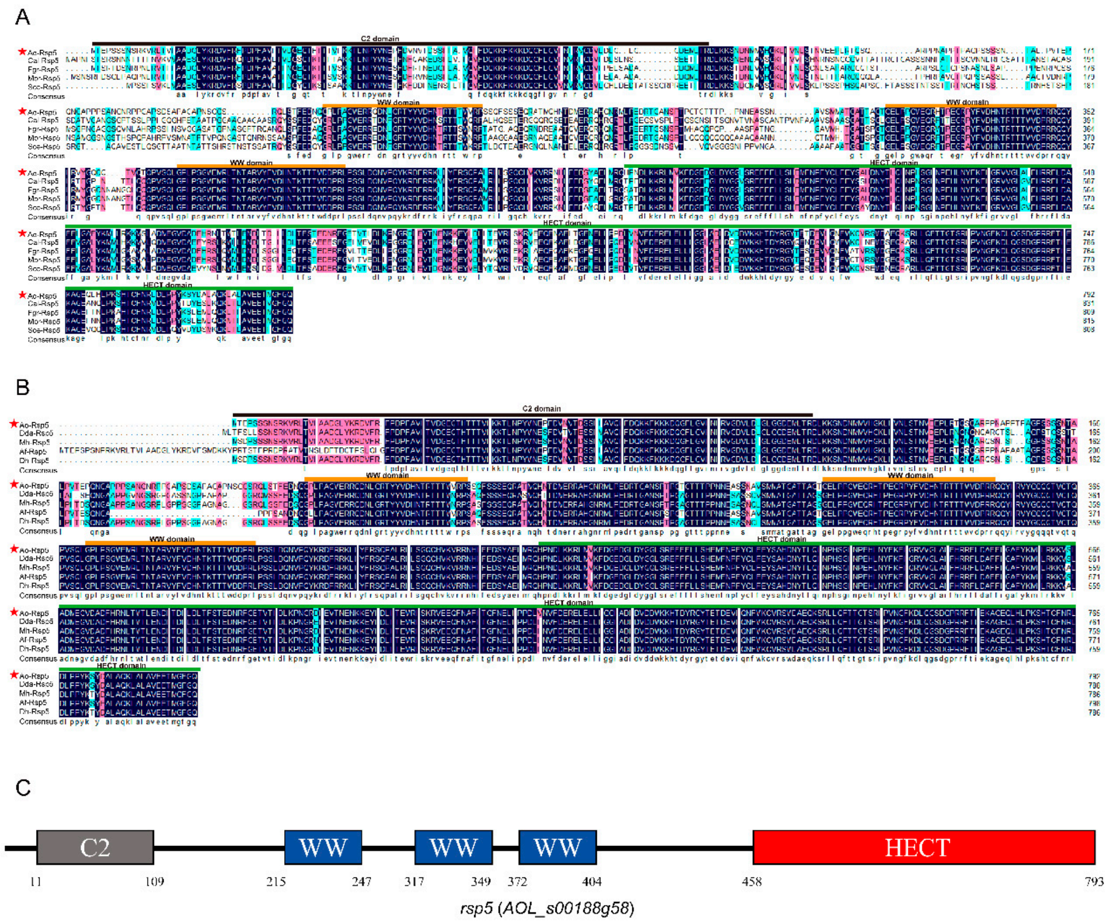

Figure S1. Sequence conservation and domain architecture of AoRsp5. (A) Multiple sequence alignment of AoRsp5 with Rsp5 homologs from diverse fungal species, including *Candida albicans*, *Fusarium graminearum*, *Magnaporthe oryzae*, and *Saccharomyces cerevisiae*. Identical residues are shaded in dark blue, and conserved residues are highlighted in light colors. Conserved functional domains (C2, WW, and HECT) are annotated with black, orange, and green lines, respectively. (B) Multiple sequence alignment of AoRsp5 with Rsp5 homologs from other nematode-trapping fungi, including *Drechslerella dactyloides*, *Monacrosporium haptotylum*, *Arthrobotrys flagrans*, and *Dactylellina haptotyla*, reveals strikingly high sequence identity across the entire protein, particularly within the core functional domains (C2, WW repeats, and the HECT catalytic domain). Residue conservation is colored as in panel (A). Red asterisks mark AoRsp5 in both alignments. (C) Schematic representation of the domain architecture of AoRsp5 in *Arthrobotrys oligospora*. The N-terminal C2 domain (residues 11–109), three tandem WW domains (residues 215–247, 317–349, 372–404), and the C-terminal HECT catalytic domain (residues 458–793) are represented by boxes.

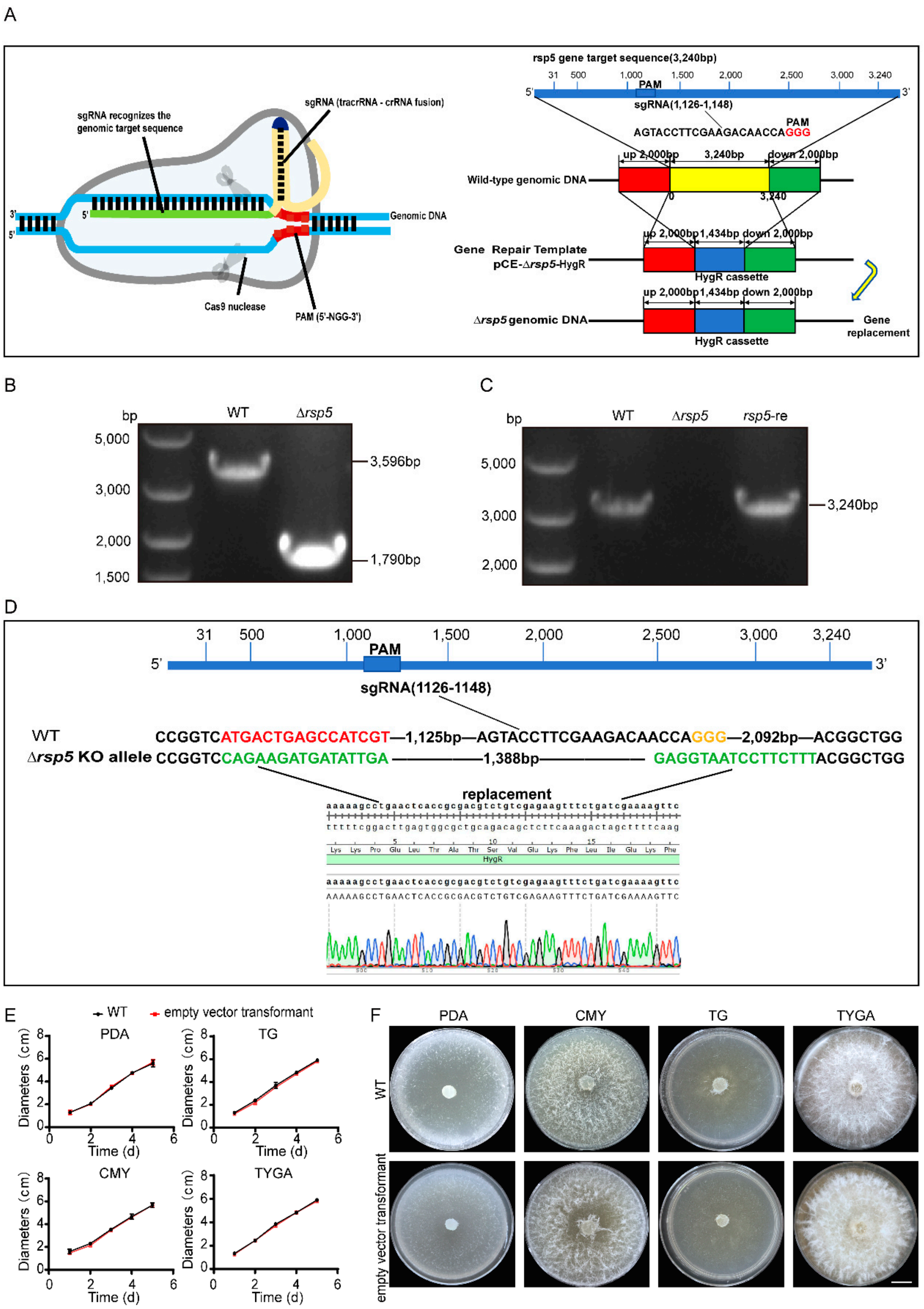

Figure S2. CRISPR-Cas9 ribonucleoprotein (RNP)-mediated homology-directed repair (HDR) for targeted deletion of ***rsp5*** and validation of the knockout strain. (A) Schematic overview of the RNP-HDR gene replacement strategy. The sgRNA targeting the *rsp5* locus (positions 1,126–1,148, with the 5′-NGG-3′ PAM motif) directs the Cas9 ribonucleoprotein complex to induce a site-specific double-strand break in the wild-type (WT) genome. The *rsp5*-KO repair fragment, containing 2000 bp upstream and 2000 bp downstream homologous arms flanking the hygromycin resistance cassette (*HygR*), mediates HDR to

replace the rsp5 coding region with the resistance marker. (B, C) Agarose gel electrophoresis of PCR-based validation. (B) Primers targeting the *rsp5* coding region amplify a 3,596 bp fragment in the WT strain and a 1,790 bp fragment in the Δ*rsp5* mutant, confirming successful gene replacement. (C) Primers spanning the recombination junction amplify a 3,240 bp fragment in the WT and complemented (*rsp5*-re) strains, while no product is detected in the Δ*rsp5* mutant, verifying targeted knockout. (D) Sanger sequencing confirmation of the *rsp5* locus. Alignment of the WT and Δ*rsp5* allele sequences shows precise replacement of the sgRNA-targeted region (including the PAM motif) with the *HygR* cassette in the knockout strain, as confirmed by the corresponding sequencing chromatogram. (E-F) Colony diameter measurement and colony images of WT and empty vector transformant strains.

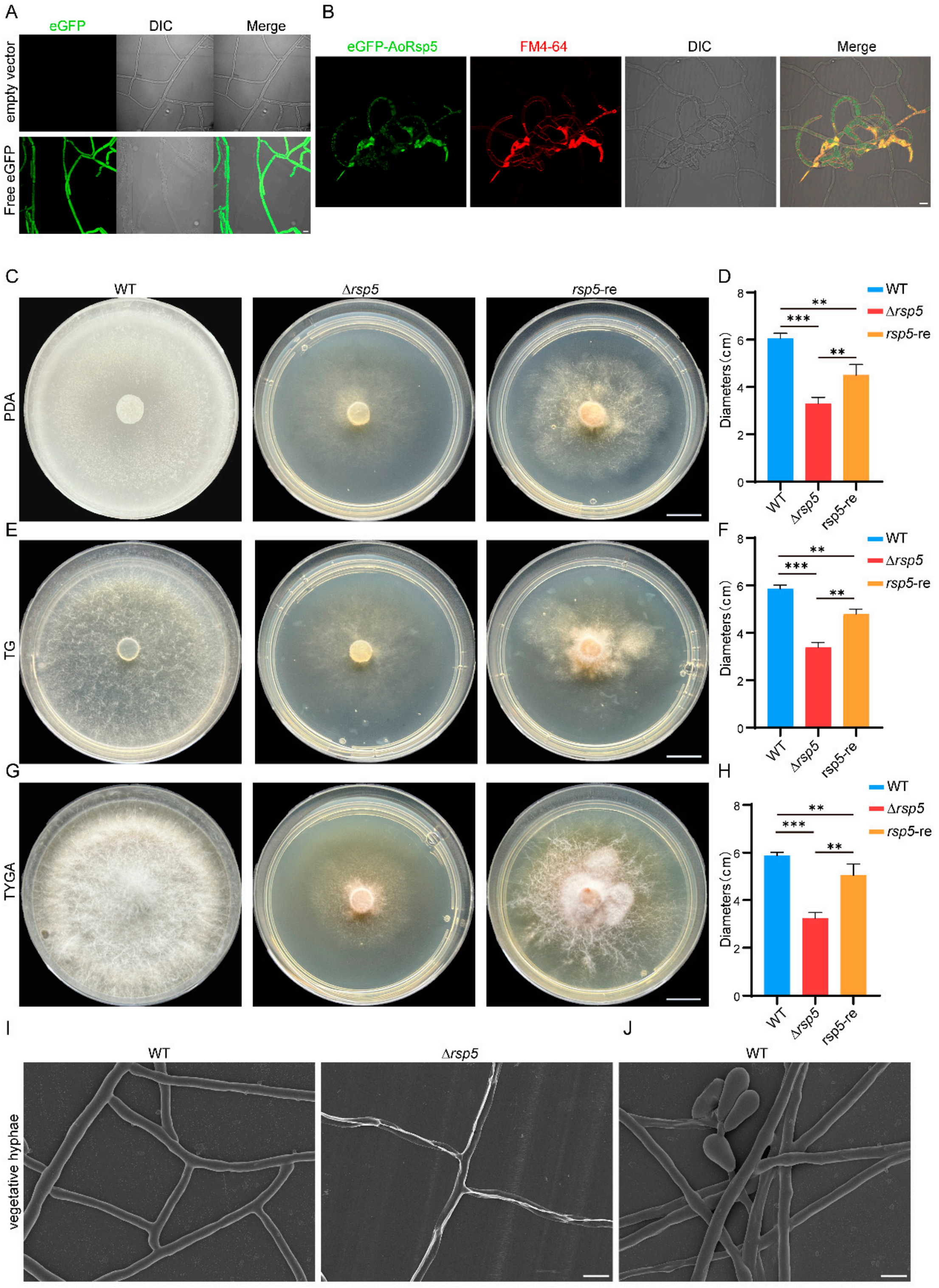

Figure S3. Subcellular localization of AoRsp5 and phenotypic characterization of the ***Δrsp5*** mutant in ***A. oligospora***. (A) Fluorescence controls: the empty vector transformant shows no eGFP signal; free eGFP exhibited diffuse cytoplasmic fluorescence throughout hyphae, lacking specific signals. Scale bar = 5 μm. (B) Subcellular localization of eGFP-AoRsp5 in *A. oligospora*. The eGFP-AoRsp5 fusion protein (green) colocalizes with the endocytic tracer FM4-64 (red) at the plasma membrane and endosomal compartments, as shown in the merged image. Scale bar = 5 μm. (C–H) Colony growth of the wild-type (WT), Δ*rsp5*, and

complemented (*rsp5*-re) strains on PDA, TG, and TYGA media. Representative colony morphologies are shown, alongside statistical comparisons of colony diameters measured after 7 days of incubation (***P* < 0.01, ****P* < 0.001; one-way ANOVA with Tukey's post-hoc test). (I) SEM analysis of hyphal morphology. Scale bars = 10 μm. (J) Conidia formation in the WT strain. Scale bars = 10 μm.

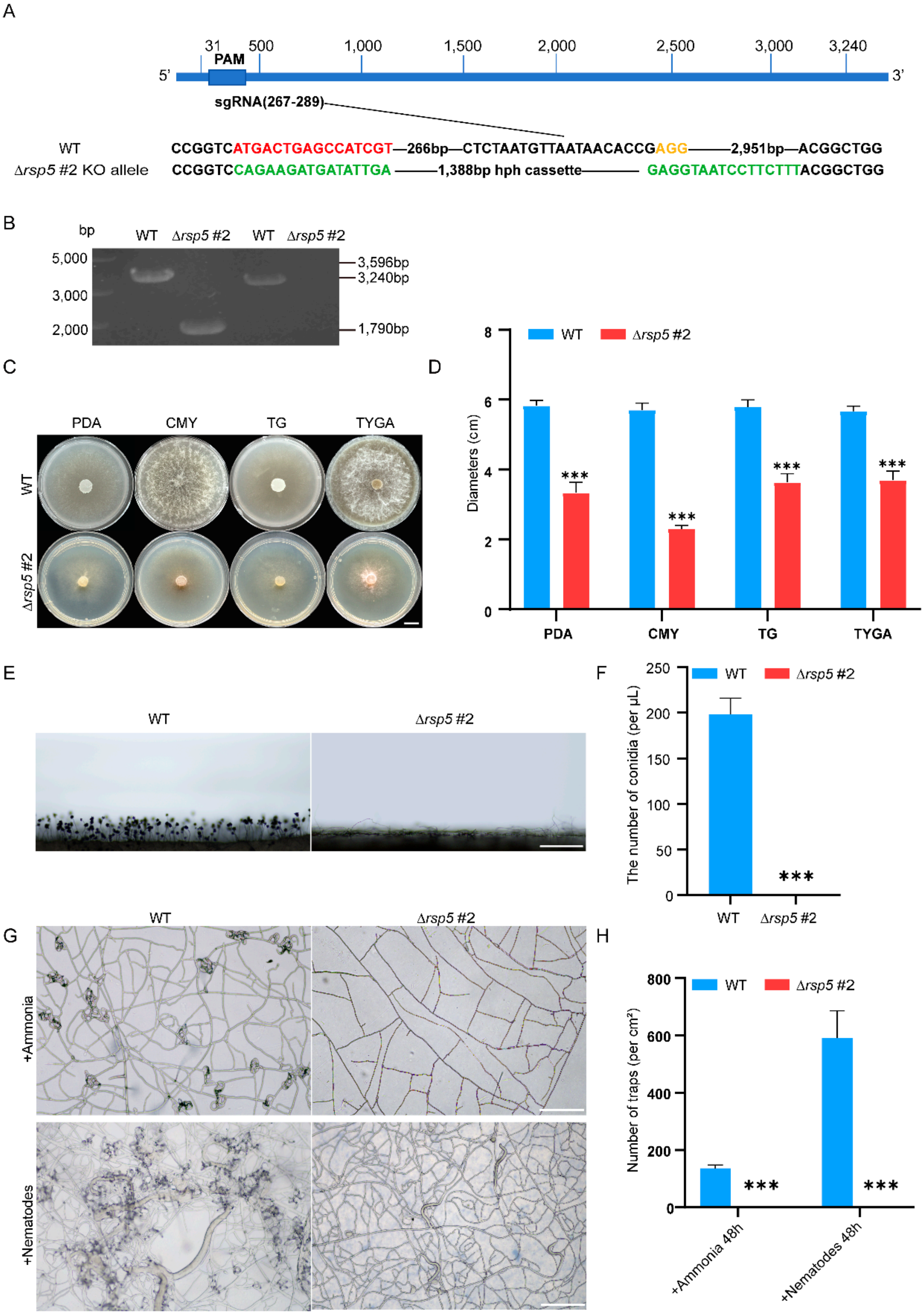

Figure S4. Phenotypic characterization of a second independent Δ*rsp5* strain (Δ*rsp5* #2) of *A. oligospora.* (A) Schematic overview of the RNP-HDR gene replacement strategy. The sgRNA targeting the *rsp5* locus

(positions 267-289, with the 5′-NGG-3′ PAM motif) directs the Cas9 ribonucleoprotein complex to induce a site-specific double-strand break in the wild-type (WT) genome. The *rsp5*-KO repair fragment, containing 2000 bp upstream and 2000 bp downstream homologous arms flanking the hygromycin resistance cassette (*HygR*), mediates HDR to replace the rsp5 coding region with the resistance marker. (B) Agarose gel electrophoresis of PCR-based validation. Primers targeting the *rsp5* coding region amplify a 3,596 bp fragment in the WT strain and a 1,790 bp fragment in the Δ*rsp5* #2, confirming successful gene replacement. Primers spanning the recombination junction amplify a 3,240 bp fragment in the WT, while no product is detected in the Δ*rsp5* #2 mutant, verifying targeted knockout. (C-D) Colony growth of the wild-type (WT) and Δ*rsp5* #2 strains on PDA, CMY, TG, and TYGA media. Representative colony morphologies are shown, together with statistical analyses of colony diameters measured at 7 days post-inoculation. (E) Microscopic observation of conidiophores in the indicated strains. Scale bar = 200 μm. (F) Quantification of conidial production in the indicated strains. (G) Microscopic observation of traps. Scale bars = 200 μm. (H) Quantification of trap formation in the indicated strains at different hours post exposure (hpe). All pairwise comparisons between WT and Δ*rsp5* #2 strains were performed using unpaired two-tailed Student's t-tests. ***P < 0.001.

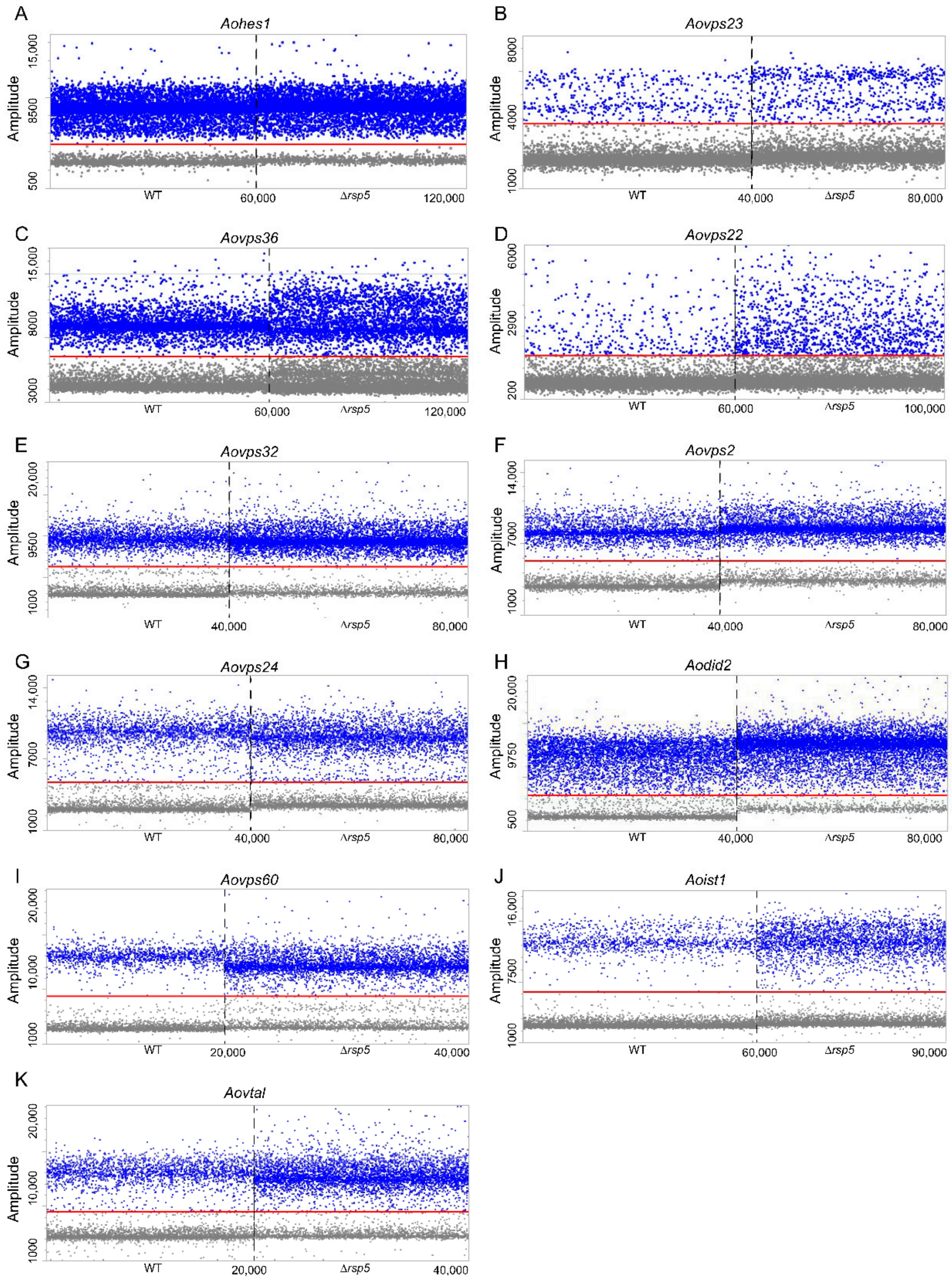

Figure S5. Digital PCR validation of **core** ESCRT pathway genes in the WT and Δ*rsp5* strains. (A-K) Absolute transcript quantification of core ESCRT subunits and functional genes via ddPCR. Thresholds are indicated by red lines.

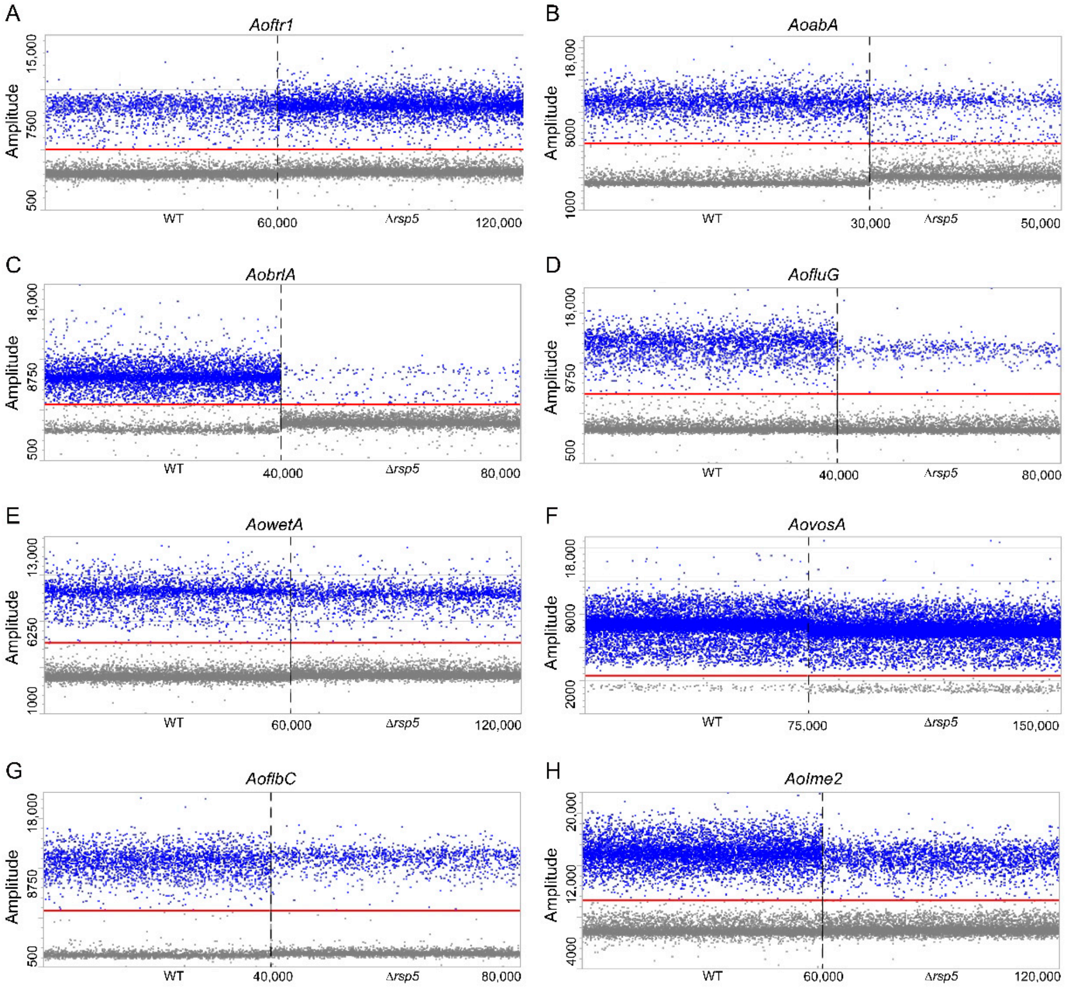

Figure S6. Digital PCR absolute transcript quantification of iron metabolism, sporulation-related, and non-canonical Ime2-type MAPK genes in WT and Δ*rsp5* strains. (A) Absolute transcript quantification of *Aoftr1* via ddPCR. (B-G) Absolute transcript quantification of sporulation-related genes via ddPCR. (H) Absolute transcript quantification of *AoIme2* via ddPCR. Thresholds are indicated by red lines.

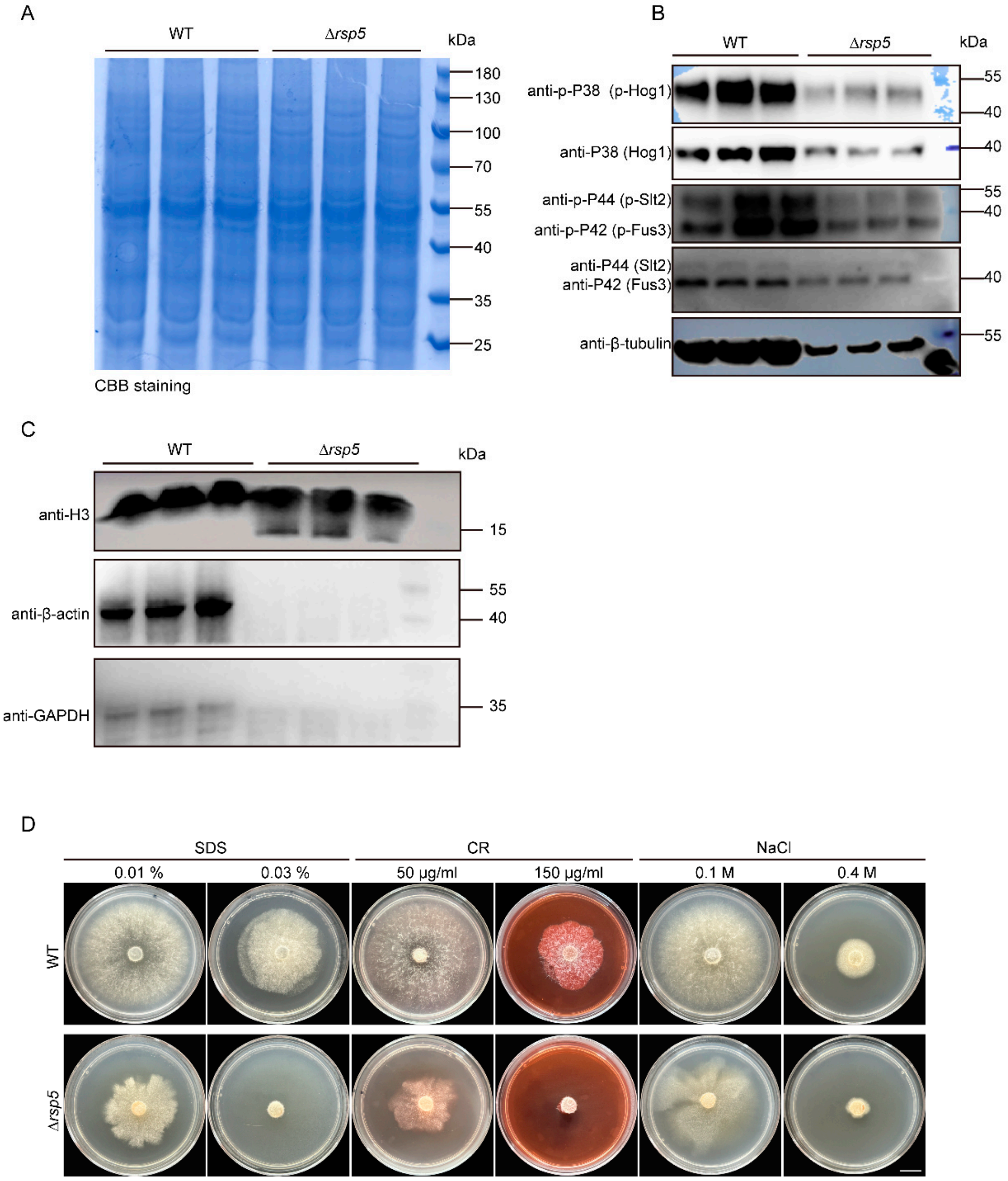

Figure S7. Total protein profiling and stress responses of WT and ***Δrsp5*** strains. (A) Coomassie blue-stained SDS-PAGE showing total protein levels in WT and Δ*rsp5*. (B) Western blots detecting total cellular ubiquitination, total cellular phosphorylation, β-tubulin, and total/phosphorylated core MAPK proteins. Coomassie staining and densitometric quantification of total protein validate equal sample loading across all lanes. (C) Western blot analysis of H3, β-actin, and GAPDH. (D) Growth of WT and Δ*rsp5* strains under cell wall and osmotic stress conditions. Scale bars = 1 cm.

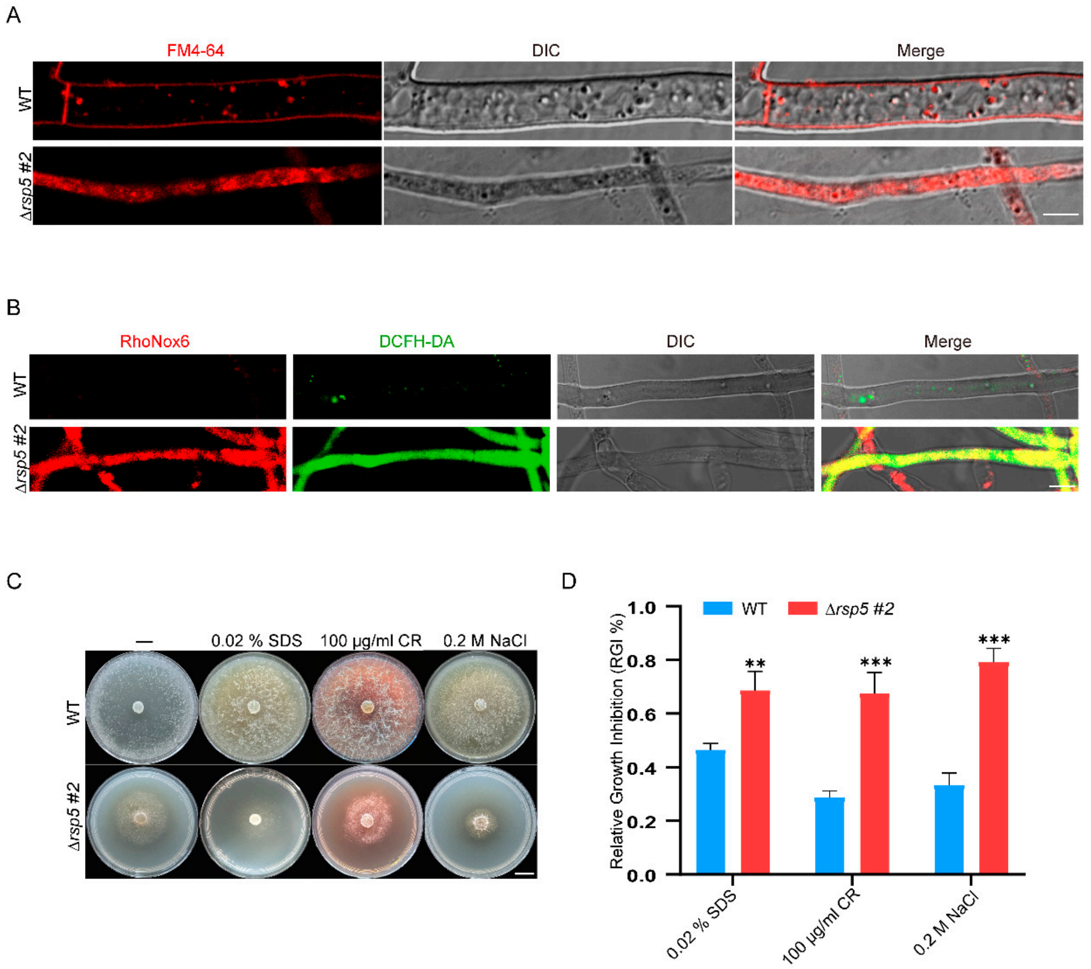

Figure S8. Phenotypic characterization of FM4-64 staining, ferroptosis, and stress resistance in the second independent Δ*rsp5* knockout strain (Δ*rsp5* #2) of *A. oligospora*. (A) FM4-64 staining for endocytic trafficking assessment. Scale bar = 5 μm. (B) RhoNox6 (Fe^{2+}) and DCFH-DA (ROS) dual staining combined with quantitative fluorescence analysis to characterize ferroptosis-like features in WT and Δ*rsp5* #2 strains. (C) Stress sensitivity assays under cell wall and osmotic stress conditions. Scale bar = 1 cm. (D) Quantification of relative growth inhibition (RGI) of WT and Δ*rsp5* #2 under each stress condition. $^{**}P < 0.01$, $^{***}P < 0.001$. All P-values were calculated using an unpaired, two-tailed Student's t-test.

Table S1. Absolute quantification of gene expression in *A. oligospora* WT and Δ*rsp5* strains by ddPCR. All reactions (20 μL) were analyzed via CH1 channel; >10,000 droplets per sample met ddPCR QC criteria. [a]Fold Change was calculated as the concentration ratio of Δrsp5 / WT, with WT set to 1.0. [b-c]Regulation

gene	Sample Name	Mean Total Droplets	Mean Positive Droplets	Mean Concentration (copies/μL)	Mean Fold Change[a] (Δrsp5/WT)
Aohse1	WT	71,029	60,002	9,117.68	1.46
	Δ*rsp5*	72,641	68,752	13,300.22	
Aovps27	WT	37,797	9,352	1,323.36	7.35 ↑[b]
	Δ*rsp5*	43,418	37,161	9,724.77	
Aovps23	WT	48,783	540	53.132	2.12 ↑
	Δ*rsp5*	41,509	898	112.6226	
Aovps28	WT	46,372	10,640	1,184.23	1.74
	Δ*rsp5*	36,542	14,349	2,057.28	
Aovps25	WT	61,598	20,555	1,890.17	2.16 ↑
	Δ*rsp5*	64,996	37,983	4,087.56	
Aovps36	WT	65,976	12,055	1,565.88	1.09
	Δ*rsp5*	59,827	19,824	1,705.73	
Aovps22	WT	52,243	604	50.319	5.03 ↑
	Δ*rsp5*	52,199	2,968	253.3095	
Aovps32	WT	35,104	12,982	2,097.88	4.28 ↑
	Δ*rsp5*	46,338	38,922	8,968.78	
Aovps2	WT	37,954	14,722	2,464.08	2.78 ↑
	Δ*rsp5*	44,028	34,291	6,855.49	
Aovps24	WT	34,714	6,919.00	1,009.94	2.38 ↑
	Δ*rsp5*	37,215	15,011	2,404.28	
Aovps20	WT	25,570	2,019	382.92	5.53 ↑
	Δ*rsp5*	19,662	7,755	2,118.98	
Aodid2	WT	43,521	24,023.00	3,474.39	3.28 ↑
	Δ*rsp5*	43,705	40,379	11,417.08	
Aovps60	WT	20,082	2,694	891.9107	6.55 ↑
	Δ*rsp5*	27,616	18,482.00	5,844.65	
Aoist1	WT	65,244	2,007	165.05	4.95 ↑
	Δ*rsp5*	53,006	7,597	817	
Aovta1	WT	24,148	3,915	984.34	5.18 ↑
	Δ*rsp5*	25,019	16,660	5,103.80	
Aovps4	WT	67,987	32,542	3,269.75	1.70
	Δ*rsp5*	68,763	45,987	5,546.98	
Aotubulin	WT	60,616	4,464	331.0108	12.72 ↑
	Δ*rsp5*	46,068	28,658	4,211	
Aoftr1	WT	63,657	4,068	248.0778	7.56 ↑
	Δ*rsp5*	67,629	25,636	1,875.38	
Aofet3	WT	46,893	4,122	379.5714	5.61 ↑
	Δ*rsp5*	43,702	16,678	2,130.62	
Aogpx	WT	60,266	22,471	2,227.17	4.71 ↑
	Δ*rsp5*	55,461	49,622	10,480.08	
AoBrlA	WT	31,066	25,174	7,739.84	0.01 ↓[c]
	Δ*rsp5*	31,558	224	39.6402	
AoabaA	WT	33,880	11,139	1,725.03	0.12 ↓
	Δ*rsp5*	22,739	1,057	205.9675	
AowetA	WT	65,085	13,289.00	1,146.49	0.61
	Δ*rsp5*	61,149	8,342	700.0943	
AoFluG	WT	41,192	7,681	914.7621	0.08 ↓
	Δ*rsp5*	36,541	584	73.1992	
AoFlbc	WT	34,816	6,537.00	1,220.41	0.25 ↓
	Δ*rsp5*	38,249	1,910.00	308.7756	
AovosA	WT	73,152	73,001	24,333.00	0.82
	Δ*rsp5*	83,029	82,310	20,063.73	
AoIme2	WT	71,221	32,062	53,028.14	0.16 ↓
	Δ*rsp5*	70,352	6,426	8,703.78	

criteria: Fold Change > 2.0 and $P < 0.05$, significant upregulation (↑); Fold Change < 0.5 and $P < 0.05$, significant downregulation (↓); $0.5 \leq$ Fold Change ≤ 2.0 or $P \geq 0.05$, no significant change.

Table S2. Primers list

	Sequence	Product length (bp)	Usage
Aorsp5-Upstream	GGATCTTCCAGAGATGCCCCAAGACCAAGA<br>TCAATATCATCTTCTGGACCGGGAGCACGA	2,031	RNP-mediated gene knockout
Aorsp5-Downstream	AGAGGTAATCCTTCTTTACGGCTGGAAGAT<br>CTGCCGTTCGACGATTTTGACGGTATAATAT	1,448	
hph	TTTCGTGCTCCCGGTCCAGAAGATGATATTG<br>TGTATCTTCCAGCCGTAAAGAAGGATTACC	2,153	
Aorsp5-HDR repair template	GCCCCAAGACCAAGAATAAAT<br>TTTGACGGTATAATATTTATA	5537	HDR-repair template PCR
Aorsp5-1	ACCTTACCCGACTTCCTTGA<br>AGGAAGAAAAAGAGAGAC	WT, 3,596;<br>Mutant, 1,790	genome PCR
Aorsp5-2	ATGACTGAGCCATCGTCAAG<br>CTCTTGTCCGAATCCCATCGTCT	WT/re, 3,240;<br>Mutant, -	
Complementation/eGFP tagging vector	TCGTCGAACGGCAGTTGAATT<br>CAGTATAGCGACCAGCATTCACA	6,641	Cloning primer
Aorsp5-3	CATCACATCACAATCGATCCATGACTGAGC<br>CCTCGCCCTTGCTCACCATCTCTTGTCCGAA	2,379	
Aorsp5-4	CTCGAGAAAAGTCAGACTAACAG<br>GTATGTGTTTGTTCCCCATC	114	RT-qPCR
Aotubulin-1	AAGGATGGAGGACAACAACGA<br>CCTTCTTTTCGTCCGTTTCCT	167	
Aohse1	CAACCCTTTTGACGATGTCGTC<br>ACGACTTCTCTCGGAGCATCT	126	ddPCR
Aovps27	GAACAGATCGAGAAGGCCAC<br>CTTGTTGCCTATCCTCCGTTTGAGAGA	135	
Aovps23	CCCAAAACCGACGTCTACAG<br>CCCAGATGTTAAGGGGGATG	122	
Aovps28	CATTGGGCAATGGACTCCAGAG<br>CATCCGCAGTCAGTTCAGAGCT	149	
Aovps25	TCTCAACCTCGCCGATACAATGGAAACTGA<br>TTTACAAGGTCGTCGACGACTTCGCGGATT	105	
Aovps36	TGCCTAGTGAAACCCTCCTCTT<br>CGAAAACTTTCGCGGTTCATCG	146	
Aovps22	AAGACTCCCAAACCCGCTCTAC<br>CGGATTTCGGAGGAGTATTGGG	130	
Aovps32	GAATGTTACGGCGAATAAGAACG<br>TTCATAGCTTCGAGGGTCTGGTAGTTTAT	162	
Aovps2	TGGAATGGGCATTCGGGAAAC<br>TCTTGCTGTTCTAGTTTGGTTCTCTCCCGGT	118	
Aovps24	CAGACAATAAAAAGCTTCATCTGGG<br>TCGAGAGTTTTGAGGGTATT	122	
Aovps20	TTGAGCAAGACGGATAAACAAC<br>TTCTAGCGACATTTCCTTTTCG	144	
Aodid2	GAGAAAGCCCTATTTAACCT<br>ATATCCTGGTTTCCTTGCTT	128	
Aovps60	CAGGCTAATATGATGACGGAT<br>CTGCCATTTCATCCTGGAGTTGTTCTATTTT	139	

Table S3. Primers list

Gene	Sequence	Product length (bp)	Usage
Aoistl	ATCGTTTACTCTCCGCCCTGCATTCCAT<br>GGAGGAGCAGGATTATCGGGAATA	126	ddPCR
Aovtal	ATCATGGACATTCTCGAACAG<br>TTGGTGCATCTTCGAGCATTCT	149	
Aovps4	CCGGGTACCGGTAAGAGTTATCT<br>GGGCGTCGAGCTCATCAATGAAA	187	
Aotubulin-2	AGACTGCGGCCCCGGGTAAAAGTTGCTGAT<br>AGCAGGCTTGAACCTCTCATTTTCTG	171	
Aoftr1	CTTGATCGCCAGTGTAATCAT<br>TTTTTGTCCGACAGTGGTTTC	133	
Aofet3	CACTTGCTCCTGGCCAGAAAATGA<br>TGGGCCCTTTGGATCTCGCACTATCAAT	137	
Aogpx	GGAAGGTCGTTCTTATCGTCAACGT<br>GCCGAGGACGACCAAACCGCGATCTTGGTA	113	
AoBrlA	TTGAGGCCTCGATCCGTAGA<br>AGGTAGATGGCGCTGTTACG	102	
AoabaA	AACTTTATGCGCCTTGTCGT<br>TTGGCTAGGTGGTCTGTACG	192	
AowetA	TCTGAATCATGGTGGTCGAGGG<br>GTCCGGTCTGGTCTTCTACACT	148	
AoFluG	GATTCCAGTCCCGTGAATTC<br>GCTAAGGAGAGGATGGGCAT	189	
AoFlbC	CTCTCCGGCAAAGACAATCG<br>GTCGACTGAGGATAGTAGCT	205	
AovosA	CCAAGGCGAGGATAACACTGATGT<br>CGGGAACACCAGTTAAGACTGGTT	143	
AoIme2	GTGCTTGGATCAAGAGAGTGTCA<br>CAGAATGTTTTCTGGCTTGATATCACGG	112	
